# Eight RGS and RGS-like Proteins Orchestrate Growth, Differentiation, and Pathogenicity of *Magnaporthe oryzae*

**DOI:** 10.1371/journal.ppat.1002450

**Published:** 2011-12-29

**Authors:** Haifeng Zhang, Wei Tang, Kaiyue Liu, Qian Huang, Xin Zhang, Xia Yan, Yue Chen, Jiansheng Wang, Zhongqiang Qi, Zhengyi Wang, Xiaobo Zheng, Ping Wang, Zhengguang Zhang

**Affiliations:** 1 Department of Plant Pathology, College of Plant Protection, Nanjing Agricultural University, and Key Laboratory of Integrated Management of Crop Diseases and Pests, Ministry of Education, Nanjing, China; 2 State Key Laboratory for Rice Biology, Biotechnology Institute, Zhejiang University, Huajiachi Campus, Hangzhou, China; 3 Department of Pediatrics and the Research Institute for Children, Louisiana State University Health Sciences Center, New Orleans, Louisiana, United States of America; University of Melbourne, Australia

## Abstract

A previous study identified MoRgs1 as an RGS protein that negative regulates G-protein signaling to control developmental processes such as conidiation and appressorium formation in *Magnaporthe oryzae*. Here, we characterized additional seven RGS and RGS-like proteins (MoRgs2 through MoRgs8). We found that MoRgs1 and MoRgs4 positively regulate surface hydrophobicity, conidiation, and mating. Indifference to MoRgs1, MoRgs4 has a role in regulating laccase and peroxidase activities. MoRgs1, MoRgs2, MoRgs3, MoRgs4, MoRgs6, and MoRgs7 are important for germ tube growth and appressorium formation. Interestingly, MoRgs7 and MoRgs8 exhibit a unique domain structure in which the RGS domain is linked to a seven-transmembrane motif, a hallmark of G-protein coupled receptors (GPCRs). We have also shown that MoRgs1 regulates mating through negative regulation of Gα MoMagB and is involved in the maintenance of cell wall integrity. While all proteins appear to be involved in the control of intracellular cAMP levels, only MoRgs1, MoRgs3, MoRgs4, and MoRgs7 are required for full virulence. Taking together, in addition to MoRgs1 functions as a prominent RGS protein in *M. oryzae*, MoRgs4 and other RGS and RGS-like proteins are also involved in a complex process governing asexual/sexual development, appressorium formation, and pathogenicity.

## Author Summary

Regulator of G-protein signaling (RGS) proteins play pivotal roles in modulating heterotrimeric G-protein signal transduction cascades that enable eukaryotic cells to perceive external cues and elicit appropriate physiological and biochemical responses. A previous study for one of the RGS proteins, MoRgs1, has demonstrated that these important roles also exist in the rice blast pathogen *Magnaporthe oryzae*. To further study functions of RGS proteins in this pathogen, we have characterized additional seven RGS and RGS-like proteins. This has been the largest number of RGS and RGS-like proteins ever discovered from a single fungal organism. We provided evidence to demonstrate that, in addition to MoRgs1 functioning as a prominent RGS protein, other RGS and RGS-like proteins are also involved in a complex process to control asexual/sexual development, appressorium differentiation and penetration, and pathogenicity of *M. oryzae*.

## Introduction

Signal transduction cascades are the primary means by which external cues are communicated to the nuclei of eukaryotic organisms including fungi. Heterotrimeric guanine-nucleotide binding protein (G-protein) signaling is one of the most important mechanisms by which eukaryotic cells sense extracellular signals and integrate them into intrinsic signal transduction pathways, such as the cyclic AMP (cAMP)-dependent signaling pathway. Heterotrimeric G-proteins are activated by the seven-transmembrane-spanning family of receptors [1]. Binding of signal ligands to such receptors promotes an exchange of GDP to GTP on the Gα subunit, which then triggers a reciprocal conformational change and dissociation from the Gβγ heterodimer [2]. Either Gα or Gβγ, or both, are then free to activate downstream target effectors such as phosphodiesterase, protein kinases, adenylyl cyclases, phospholipases, and ion channels [3]–[6]. The activated G-proteins are later desensitized by the intrinsic GTPase activity of the Gα subunit, followed by re-association with the Gβγ complex. Therefore, the guanine nucleotide state of the Gα subunit plays a critical role in controlling G-protein signaling [2]. In fungi, G-proteins are involved in the regulation of a variety of cellular functions in vegetative growth and/or pathogenic development, such as conidiation, infection structure differentiation, and pathogenicity [7]–[9].

Regulators of G-protein signaling (RGS) proteins primarily function as GTPase-accelerating proteins (GAPs) that promote GTP hydrolysis by the Gα subunits, thereby inactivating the G-protein and rapidly switching off G protein-coupled signaling pathways [10], [11]. All RGS proteins contain a conserved domain of ∼120 amino acids that are required for activity and function as key negative regulators of G-protein signaling pathways [12]–[14]. The budding yeast *Saccharomyces cerevisiae* contains four RGS and RGS-like proteins: Sst2, Rgs2, Rax1, and Mdm1. The archetypical RGS protein Sst2 possesses two N-terminal DEP (*D*isheveled, *E*GL-10, *P*leckstrin) homology domains and a C-terminal RGS domain, Rgs2 has an N-terminal RGS domain, Rax1 has an N-terminal RGS domain and three C-terminal trans-membrane motifs, and Mdm1 contains an N-terminal PXA and a C-terminal PX domain in addition to an RGS domain [15].

The ascomycete *Magnaporthe oryzae* is pathogenic to important crops such as rice, barley, wheat, and millet. Rice blast, caused by this heterothallic haploid fungus, is one of the most severe fungal diseases of rice throughout the world [16]. Genetic studies of this important pathogen have advanced dramatically in the past decade, and thus it is an excellent model system for investigating plant–pathogen interactions. *M. oryzae* infects rice plants in a manner typical of many other foliar pathogens. Germ tubes produced from conidia attached to leaf surfaces differentiate into specialized infection structures called appressoria. The enormous turgor pressure generated in appressoria by the accumulation of high concentrations of glycerol is used to penetrate the underlying plant surface [17]. Mutants blocked at appressorium formation or appressorial turgor generation fail to infect healthy rice plants [18]. After penetration, infection hyphae grow in and between plant cells, and eventually result in lesion formation on the plant. Thousands of conidia are produced on the lesions and then released to initiate a new disease cycle on new plant tissues within 3–5 days. Initiation of appressorium formation in *M. oryzae* was shown to require G-protein and cAMP signaling, because loss of Gα MoMagB and adenylyl cyclase MoMac1 leads to failure in appressorium formation [19], [20]. A MAP kinase cascade has also been identified as an essential signaling pathway involved in appressorium formation during pathogenic development [21]–[23]. *M. oryzae* contains three distinct Gα proteins (MoMagA, MoMagB, and MoMagC), two Gβ subunits (MoMgb1 and MoMgb2), and one Gγ subunit [19], [24], [25]. Previous studies revealed that a constitutively active allele, MoMagB^G42R^, and MoMgb1 have affected G-protein signaling in vegetative growth, sexual reproduction, and pathogenicity in *M. oryzae* [24], [26]. The Δ*Momgb1* mutant also has a defect in appressoria formation, whereas increased MoMgb1 levels promote precocious appressoria formation [24]. Moreover, expression of a dominant active allele of *MoMAGB* caused appressoria to form on non-inductive surfaces, while exogenous cAMP can activate appressorium formation in a Δ*MomagB* mutant [19], [26], [27], indicating that MoMagB may sense surface cues and stimulate cAMP synthesis. The regulator of G protein signaling Rgs1, which interacts with all three Gα subunit, was shown to negatively regulate G-protein signaling. Deletion of *MoRGS1* leads to a significant increase in intracellular cAMP levels and conidiation, and Δ*Morgs1* mutants also form appressoria on non-inductive hydrophilic surfaces [28]. These observations suggest that G-protein signaling and its regulators play important roles in activating the downstream cAMP pathway and regulating vegetative growth and pathogenic development. Further characterization of G-protein regulators will be helpful in better understanding the role of G-protein-mediated signaling in the regulation of early events during plant infection by the rice blast fungus.

Here, we systematically characterized all eight RGS proteins (MoRgs1–8) in *M. oryzae*. We found MoRgs1 has a role in regulating cell wall integrity and surface hydrophobicity, in addition to a role in mycelia growth, conidiation, sexual reproduction, and pathogenicity as previously reported [28]. All RGS proteins were involved to certain degree in the regulation of intracellular cAMP levels. Other RGS proteins, MoRgs4 in particular, also exhibit various degree of roles in conidiation, vegetative growth, asexual and sexual development, appressorium formation, and pathogenicity.

## Results

### *M. oryzae* contains eight genes encoding RGS and RGS-like proteins

MoRgs1 was first identified as a negative regulator of the G-protein signaling pathway during the important developmental events such as conidial and appressorium formation in *M. oryzae* [28]. To obtain a comprehensive understanding of RGS protein functions, seven additional genes encoding RGS and RGS-like proteins were identified and their biological functions characterized.

**Figure 1:**
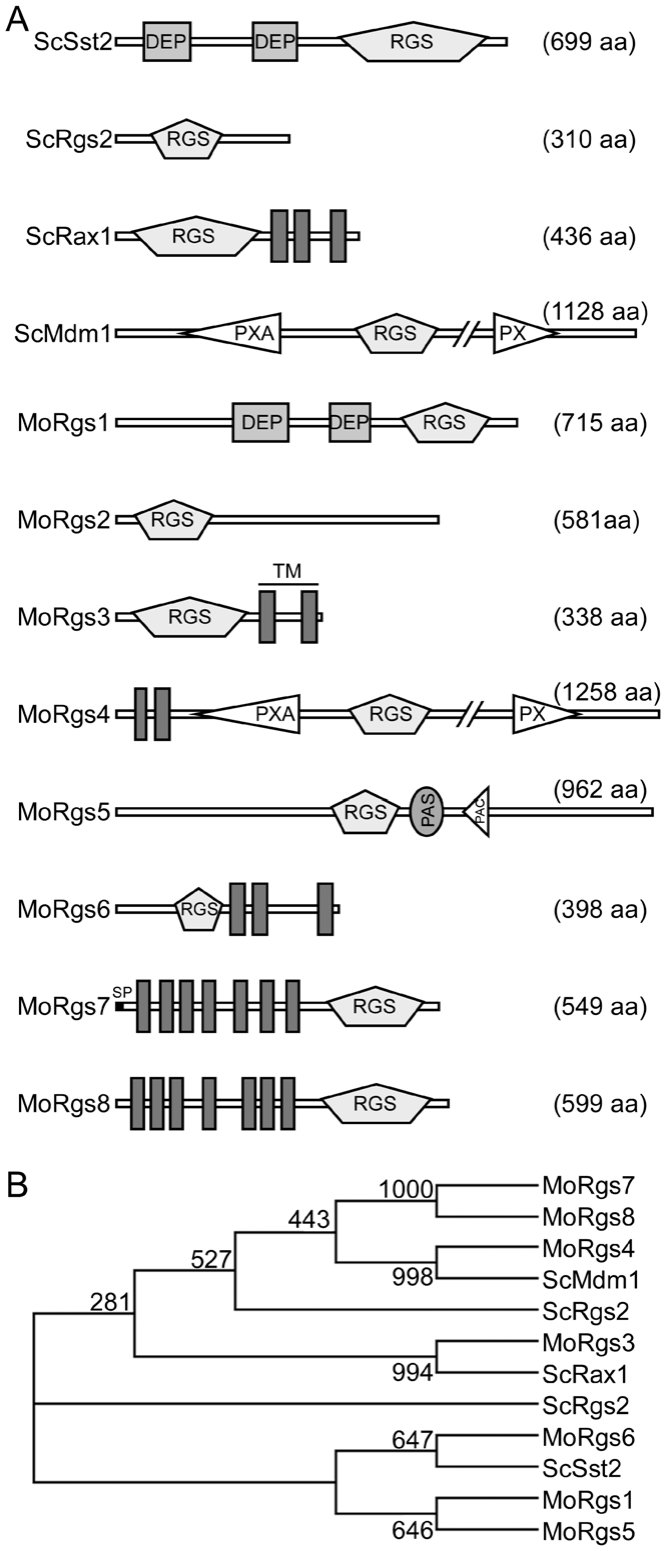
*M. oryzae* encodes eight RGS and RGS-like proteins. (A) Schematic representation of all eight *M. oryzae* RGS proteins and their comparison to those of *S. cerevisiae*. DEP, domains found in Dishevelled, Egl-10, and pleckstrin; PX, domains that bind to phosphoinositides; TM, transmembrane; aa, amino acids; SP, signal peptide. (B) The alignment of *M. oryzae* (Mo) and *S. cerevisiae* (Sc) RGS proteins indicates higher amino acid sequence similarity between homologs. Protein sequences were aligned, and the phylogenic tree was drawn using Clustal W 1.83. The GenBank accession numbers are as follows: MoRgs1, ABC60049; MoRgs2, XP_361183; MoRgs3, XP_360603; MoRgs4, XP_368254; MoRgs5, XP_363151; MoRgs6, XP_364773; ScSst2, NP_013557; ScRgs2, NP_014750; ScRax1, NP_014945; ScMdm1, NP_013603; MoRgs7, XP_001411659; MoRgs8, XP_001405673.

*S. cerevisiae* Sst2, Rgs2, Rax1, and Mdm1 RGS protein sequences were used to search the *M. oryzae* genome database using blastp (http://www.broadinstitute.org/annotation/genome/magnaporthe_grisea-/MultiHome.html), and each yielded a single homolog named MoRgs1 (MGG_14517.6), MoRgs2 (MGG_03146.6), MoRgs3 (MGG_03726.6), and MoRgs4 (MGG_00990.6), respectively. As expected, MoRgs1 remains as the same as previously described [28], whereas MoRgs2, MoRgs3, and MoRgs4 share high similarities to *S. cerevisiae* Rgs2, Rax1, and Mdm1 in the domain architectures with the amino acid sequence identity within the RGS domain being 26%, 26%, and 19% respectively (Figure 1A). In addition to these proteins, further search led to the identification of MoRgs5 (MGG_08735.6), MoRgs6 (MGG_09618.6), MoRgs7 (MGG_11693.6), and MoRgs8 (MGG_13926.6). Amino acid sequence similarities between each homolog are shown through phylogenetic analysis (Figure 1B). MoRgs5 contains an N-terminal RGS domain and a C-terminal PAS and PAC domain, while MoRgs6 possesses an N-terminal RGS domain and three C-terminal transmembrane domains. MoRgs7 and MoRgs8 appear unique it that they contain multiple transmembrane domains (seven) N-terminus of the RGS domain (Figure 1C).

### Expression patterns reveal potential roles of RGS proteins in conidiogenesis, appressorium development, and infectious growth

To gain insight into the possible functions of these RGS and RGS-like proteins, we examined the gene transcription profiles during conidial stages by quantitative real-time PCR (qRT-PCR). Compared to the mycelium stage, the transcription of all *MoRGS* genes was upregulated; expression levels of *MoRGS1* (>50-fold), *MoRGS2* (>48-fold), *MoRGS3* (> 44-fold), *MoRGS6* (> 82-fold), *MoRGS7* (> 88-fold), and were much higher than those of *MoRGS4* (> four-fold), *MoRGS5* (> seven-fold), and *MoRGS8* (> six-fold). In the appressorium stage, *MoRGS1* to *MoRGS6* genes also showed high transcription levels than those during the mycelial stage. Besides *MoRGS2* that three-fold increase, the other five *MoRGS* genes showed significant increases in transcription (*MoRGS1*, > 59-fold; *MoRGS3*, > 19-fold; *MoRGS4*, > 31-fold; *MoRGS5*, > 11-fold; *MoRGS6*, > 87-fold) (Table 1). During the infection stage, all eight *RGS* genes also showed increased expression levels; however, only the increases in *MoRGS1*, *MoRGS3*, and *MoRGS7* were significant (*MoRGS1* > 12-fold; *MoRGS3* > 10-fold; *MoRGS3* > 17-fold), in comparison to *MoRGS2* (> three-fold), *MoRGS4* (> 1.9-fold), *MoRGS5* (> two-fold), *MoRGS6* (> four-fold), and *MoRGS7* (> two-fold) (Table 1). These results suggest that RGS proteins in *M. oryzae* likely play various roles in conidiogenesis, appressorium formation, and infection of the host plant.

**Table 1:** Real-time RT-PCR quantification of *MoRGS* gene expression in *M.oryzae*.

	Nomalized gene level relative to Actin^a^							
RNA source (Wild-type)	*MoRGS1*	*MoRGS2*	*MoRGS3*	*MoRGS4*	*MoRGS5*	*MoRGS6*	*MoRGS7*	*MoRGS8*
Mycelium	1.00(0.6–1.67)	1.00(0.95–1.05)	1.00(0.97–1.03)	1.00(0.82–1.22)	1.00(0.77–1.30)	1.00 (0.80–1.25)	1.00 (0.96–1.04)	1.00 (0.88–1.14)^b^
Conidium	58.22(53.28–63.61)	48.84(44.45–53.66)	44.22(42.12–46.43)	4.22(3.44–5.17)	7.75(6.72–8.92)	82.6(80.61–91.63)	88.49(74.75–85.31)	5.95(5.03–7.04)
Appressorium	59.58 (47.96–74.01)	3.45 (2.86–4.17)	19.65 (17.94–21.53)	31.41(30.63–32.22)	11.55 (8.66–15.41)	87.43 (73.42–104.11)	-	-
Infection stage	12.5 (9.59–16.29)	3.29 (3.02–3.58)	10.78 (9.74–11.93)	1.94 (0.84–4.47)	2.40 (1.00–5.75)	4.05 (2.84–5.76)	17.76(16.73–18.85)	2.01(1.90–2.12)

a Relative quantity of *MoRGS* genes at different developmental stages of the wild-type strain Guy11.

b The mean and range of three replicates.

### Opposed roles of MoRgs1 and MoRgs4, and MoRgs2 and MoRgs3 in asexual reproduction

**Figure 2:**
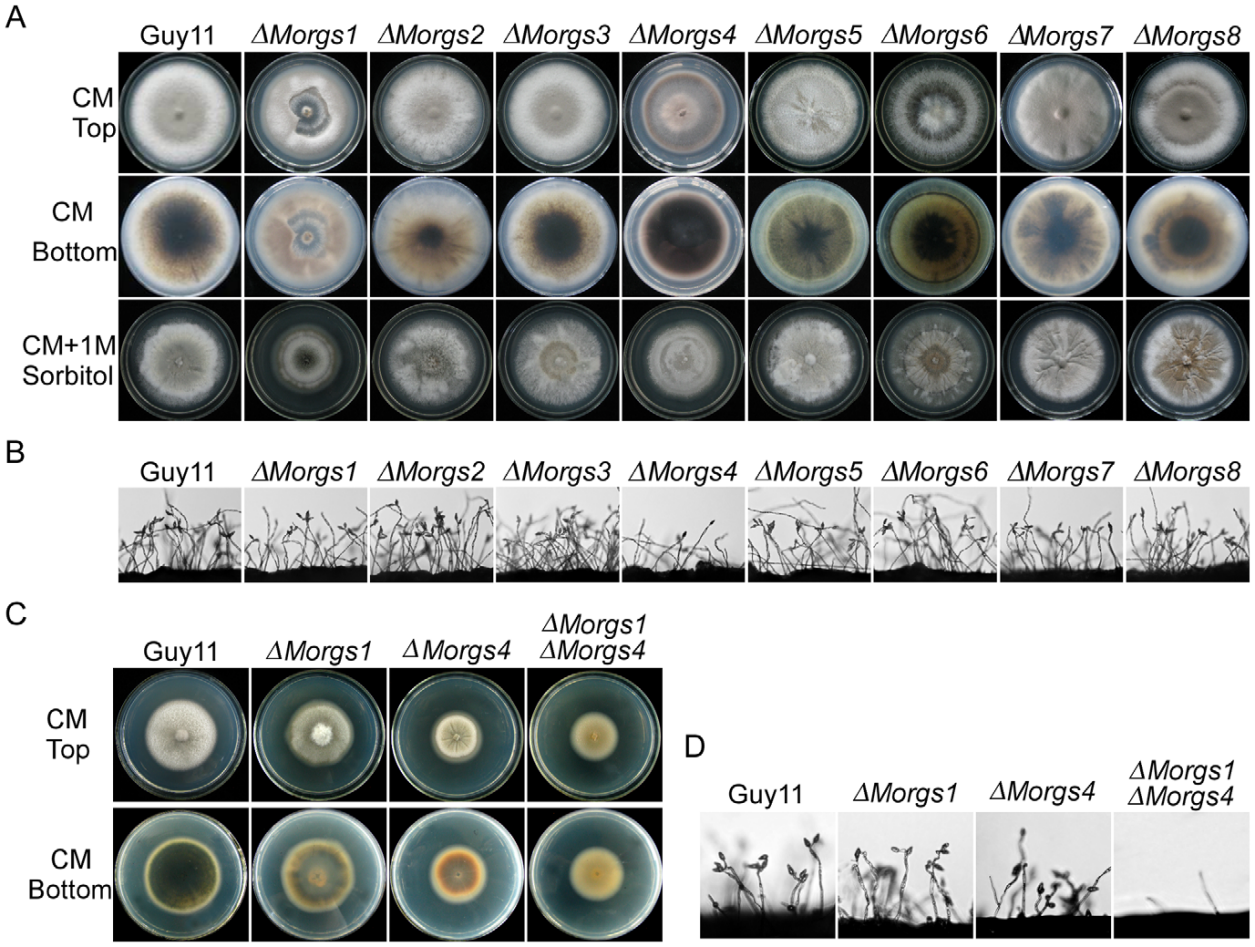
Comparison of various Δ*Morgs* mutant strains in colony morphology and conidia formation. (A) Colony morphology was observed by incubating culture plates in the dark for ten days at 28°C and then photographed. (B) Conidia formation was observed under a light microscope 24 hours at room temperature after induction of conidiation under cover slips. (C) Comparison of specific single and double mutants in colony formation in the dark for eight days at 28°C and then photographed. (D) Comparison of specific single and double mutants in conidia formation 24 hours at room temperature after induction of conidiation under cover slips.

Mutant strains specific to each RGS gene were generated and verified (Figure S1A–D). Since G-protein and the cAMP pathway are important in conidium formation and loss of MoRgs1 and phosphodiesterase MoPdeH led to enhanced conidiation [28], [29], the role of the additional RGS proteins in conidiogenesis was examined. Under normal conditions, only Δ*Morgs4* and Δ*Morgs*6 mutant strains exhibited darkened colony with less dense hyphal mat and only Δ*Morgs1* exhibited progressive autolysis (Figure 2A). Microscopic observations indicated that Δ*Morgs1* and Δ*Morgs4* produced fewer conidiophores and conidia than the wild type strain. In contrast, Δ*Morgs2* and Δ*Morgs3* showed enhanced conidiophore and conidium formation, and Δ*Morgs5*, Δ*Morgs6*, Δ*Morgs7* and Δ*Morgs8* exhibited no observable changes in the production of conidiophores and conidia (Figure 2B). To validate these findings, the numbers of conidia produced from each plate were quantified. Consistently, the conidia number of Δ*Morgs1* and Δ*Morgs4* was reduced to 0.64-fold and 0.038-fold that of the wild type, while that of Δ*Morgs2* and Δ*Morgs3* was increased 1.59-fold and 1.55-fold, and Δ*Morgs5*, Δ*Morgs6*, Δ*Morgs7* and Δ*Morgs8* strains produced nearly the same number of conidia, 1.02-fold and 1.08-fold, as the wild type, respectively (Table 2). Our finding suggests differentiated roles in conidiophore development and conidia formation by various RGS proteins in *M. oryzae*: MoRgs1 and MoRgs4 have positive roles, while MoRgs2 and MoRgs3 have negative effect on the regulation of these processes.

**Table 2:** Comparison of mycological characteristics among strains.

	Growth rate (cm)^a^					
Strain	CM	SDC	Biomass^b^ (mg)	Conidiation^c^ (×100/cm^2^)	Appressorium^d^ formation (%)	Penetration^e^ (%)
Guy11	6.5±0.2	5.9±0.1	0.1255±0.0060	99.6±18.4	96.9±6.2	85.3±3.3
*ΔMorgs1*	5.0±0.1	4.5±0.1	0.0575±0.0115	64.0±10.2	97.4±5.0	78.9±3.1
*ΔMorgs2*	6.3±0.1	5.4±0.1	0.0876±0.0163	158.3±22.4	98.0±2.4	85.6±3.9
*ΔMorgs3*	6.4±0.2	5.8±0.2	0.0728±0.0070	154.7±26.0	96.3±4.7	61.1±1.9
*ΔMorgs4*	5.5±0.1	4.5±0.1	0.0505±0.0040	3.8±1.6	98.2±6.6	85.6±4.3
*ΔMorgs5*	6.7±0.2	6.7±0.2	0.1285±0.0034	101.2±20.2	97.8±5.8	83.0±3.8
*ΔMorgs6*	6.7±0.2	6.1±0.1	0.1145±0.0065	107.5±21.8	98.7±3.0	82.4±3.2
*ΔMorgs7*	6.3±0.2	5.9±0.1	0.1001±0.0050	95.5±9.2	97.6±5.6	20.6±1.8
*ΔMorgs8*	6.5±0.2	6.5±0.2	0.1443±0.0203	97.0±15.2	99.0±8.4	84.8±4.4

a Diameter of hyphal radii at day 10 after incubation on CM and SDC agar plates at room temperature.

b Dry weight of hyphal at day 2 after incubation in liquid complete medium at room temperature by shaken at 150 rpm.

c Number of conidia harvested from a 9 cm SDC plate at day 10 after incubation at room temperature.

d Percentage of appressorium formation on artificial surface at 24 h post-inoculation at room temperature.

e Percentage of appressoria penetrated onion epidermal cells at 24 h post-inoculation.

Mean and standard deviations were calculated with results from three replicates.

To further evaluate the role of MoRgs1 and MoRgs4 in conidiation, a Δ*Morgs1* Δ*Morgs4* double mutant strain was generated and characterized (Figure S2). The mutant showed even more pronounced defect in vegetative growth with almost no conidia or conidiophores found (Figure 2C and 2D), indicating that MoRgs1 and MoRgs4 function on different targets in conidiogenesis.

### Effects of MoRgs2-8 on appressorium formation

**Figure 3:**
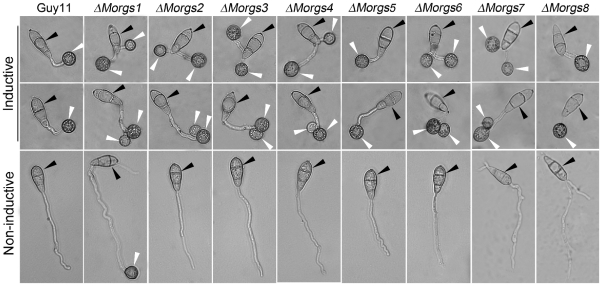
Comparison of Δ*Morgs* mutant strains in appressorium formation. Appressorium formation was allowed in either inductive or non- inductive conditions. Conidia from each strain were incubated on hydrophobic (upper two panels) and hydrophilic surfaces for 24 hours (lower panel) and photographed. Black arrows indicate spores; white arrows indicate appresoria.

In *M. oryzae*, physical cues of an inductive surface, such as hardness and hydrophobicity, are required for appressorium formation [28]. However, appressorium can be induced on non-inductive surfaces in the presence of exogenous cAMP or inhibitors of cAMP phosphodiesterase [30]. Since MoRgs1 regulates cAMP levels and the Δ*Morgs1* mutant formed normal appressoria on non-inductive surfaces [28], we studied the functions of other RGS and RGS-like proteins in appressorium formation. No appressoria formation was observed in mutant strains of Δ*Morgs2*, Δ*Morgs3*, Δ*Morgs4*, Δ*Morgs5*, Δ*Morgs6*, Δ*Morgs7*, and Δ*Morgs8* on non-inductive surfaces (Figure 3). On inductive surfaces, all of the mutant strains, except Δ*Morgs5* and Δ*Morgs8*, often produced two appressoria, either on branched germ tubes or on two germ tubes that emerged from one conidial cell after extended incubation of over 24 hours (Figure 3). The percentage of conidia forming two appressoria on germ tubes emerging from a single conidial cell was as high as 15% in these mutants. The appressoria formed on the secondary branching germ tubes were usually smaller than those formed on the primary germ tubes (Figure 3). These results indicate that, apart from MoRgs5 and MoRgs8, MoRgs2, MoRgs3, MoRgs4, MoRgs6, and MoRgs7 may also be involved in germ tube growth and appressoria formation.

### MoRgs1 and MoRgs4 are indispensable for sexual reproduction

**Figure 4:**
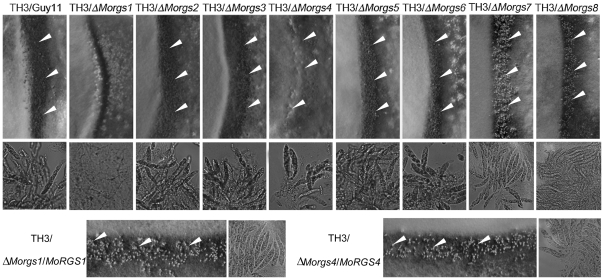
MoRgs1 and MoRgs4 are involved in sexual reproduction in *M. oryzae*. Perithecia development by wild type and Δ*Morgs* mutant strains were photographed three weeks after inoculation. Cross between TH3 (*MAT1-1*) and Guy11 (*MAT1-2*) represents the positive control. Cross of Δ*Morgs1* and Δ*Morgs4* with TH3 produced either no (Δ*Morgs1*) or less (Δ*Morgs4*) peritheria and asci. Δ*Morgs1/MoRGS1* and Δ*Morgs4/MoRGS4* indicate complement transformants. Arrows indicate peritheria.

A previous study revealed that G-protein signaling is involved in sexual development in *M. oryzae* [26]. To determine whether additional proteins play any roles in mating, the Δ*Morgs* mutant and the wild-type strains (Guy11, *MAT1-2*) were crossed to a standard tester strain, TH3 (*MAT1-1*). After 3 weeks, numerous perithecia were observed at the junctions of the cross between the wild-type strain and TH3, complement transformants (Δ*Morgs1/MoRGS1* and Δ*Morgs4/MoRGS4*)/TH3, but no or only a few perithecia were found for crosses between Δ*Morgs1* and TH3, and Δ*Morgs4* and TH3 (Figure 4, upper panel). No ascus was produced for Δ*Morgs1* x TH3 and very few asci for Δ*Morgs4* x TH3 (Figure 4, middle panel). These results suggest that MoRgs1 and MoRgs4 have positive roles in mating.

### MoRgs1 is essential for the maintenance of cell wall integrity

In our most recent study, deletion of the *MoPDEH* gene encoding phosphodiesterase resulted in a cell wall integrity defect, and the Δ*MopdeH* mutant also underwent progressive autolysis of mycelia after incubation on CM agar plates for over 14 days [31]. This is similar to autolysis occurred in mutant strains of Δ*Momps1* and Δ*Momck1* mutants [32], [33]. MoMps1 and MoMck1 are homologs of *S. cerevisiae* Slt2 and Bck1 proteins that are involved in cell wall integrity. Because RGS proteins are generally negative regulators of G-protein signaling, deletion of *RGS* genes may activate downstream cAMP signals, as occurred in the Δ*MopdeH* mutant [31]. We tested all Δ*Morgs* mutants on CM agar plates for autolysis. Only the mycelia of the Δ*Morgs1* mutant underwent progressive autolysis after incubation for 14 days, similar to the Δ*MopdeH* mutant, and none of the mutant strains showed any autolysis (Figure 2A). Moreover, the autolysis phenotype of the Δ*Morgs1* mutant was suppressed by addition of 1 M sorbitol to the culture medium (Figure 2A), as also found with the Δ*MopdeH* and Δ*Momps1* mutants [32], [33]. These results suggest that MoRgs1 plays an important role in the maintenance of cell wall integrity.

### MoRgs1 and MoRgs4 are required for surface hydrophobicity

**Figure 5:**
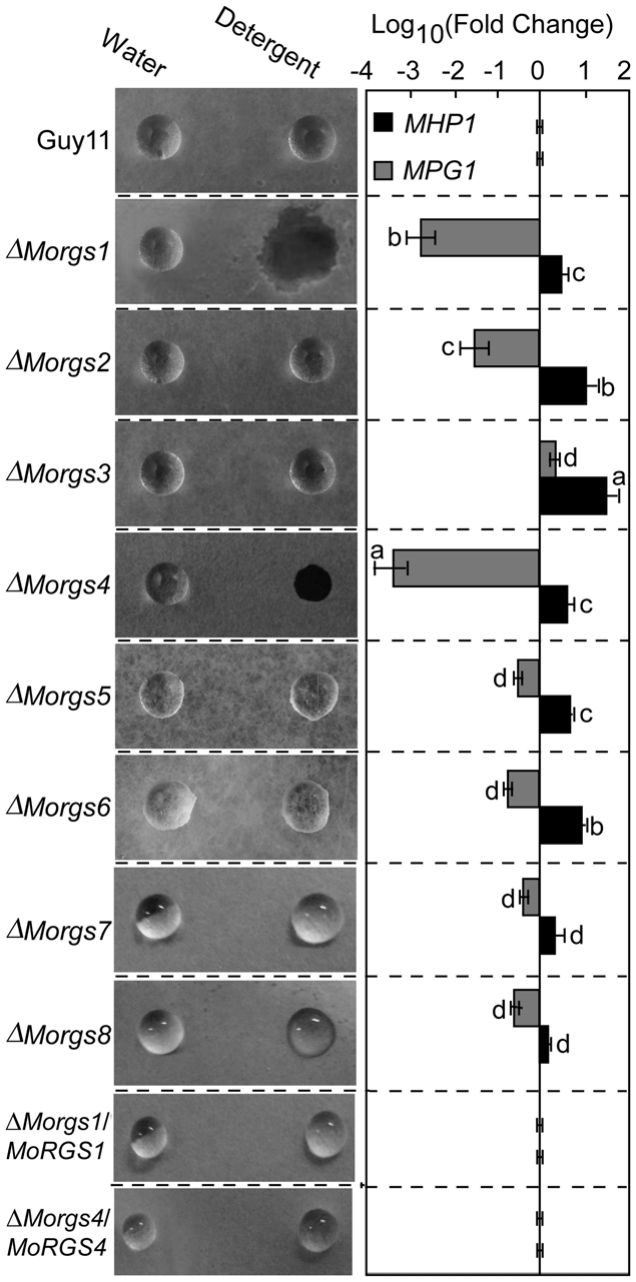
Detergent wettable phenotype of Δ*Morgs1* to Δ*Morgs8* mutants. Ten microlitres of water or detergent solution containing 0.02% SDS and 5 mM EDTA were placed on the colony surfaces of the wild type and mutant strains and photographed after 5 min (Left panel). Expression analysis of *MoMPG1* and *MoMHP1* genes in each Δ*Morgs* mutant (Right panel). Δ*Morgs1/MoRGS1* and Δ*Morgs4/MoRGS4* indicate complement transformants. The error bars indicate SD of three replicates. Different letters in each data column indicate significant differences at *P* = 0.01.

Disruption of several hydrophobin genes of *M. oryzae*, including *MoMPG1* and *MoMHP1*, and *MoPDEH* resulted in a water- or detergent-soaked, easily wettable phenotype [34], [35], [36], [37], [38], [39], [40]. To determine whether any RGS proteins are involved in surface hydrophobicity, all Δ*Morgs* mutant strains were tested with water and detergent solutions. Compared with wild type and complement transformants (Δ*Morgs1/MoRGS1* and Δ*Morgs4/MoRGS4*), none showed an easily wettable phenotype when incubated with water droplets (10 µl) after several hours. However, aerial hyphae of Δ*Morgs1* and Δ*Morgs4* mutants that were grown on CM agar were more readily wettable with a solution containing both 0.02% SDS and 5 mM EDTA within 5 min (Figure 5). This is similar to the Δ*Momhp1* and Δ*MopdeH* mutants [31]. Because the expression levels of *MoMPG1* and *MoMHP1* were altered in the Δ*MopdeH* mutant [31], we speculate that the surface hydrophobicity defect may also be related to MoMpg1 and MoMhp1. To test this hypothesis, we examined the expression levels of *MoMPG1* and *MoMHP1*. Like the Δ*MopdeH* mutant, the *MoMPG1* expression level showed a significant decrease (> 1000-fold) in the Δ*Morgs1* and Δ*Morgs4* mutants, and a relatively small decrease in the Δ*Morgs2* mutant (> 30-fold). In contrast, *MoMHP1* expression increased to different extents in all of the mutants: the fold increase for Δ*Morgs1*, Δ*Morgs2*, Δ*Morgs3*, Δ*Morgs4*, Δ*Morgs5*, Δ*Morgs6*, Δ*Morgs7*, and Δ*Morgs8* was two, 10, 30, three, three, six, two, and three-fold, respectively (Figure 5). These results indicate that MoRgs1 and MoRgs4 play a role in regulating surface hydrophobicity, likely through regulation of the *MoMPG1* expression levels.

### RGS proteins regulate *MoPTH11* expression

**Figure 6:**
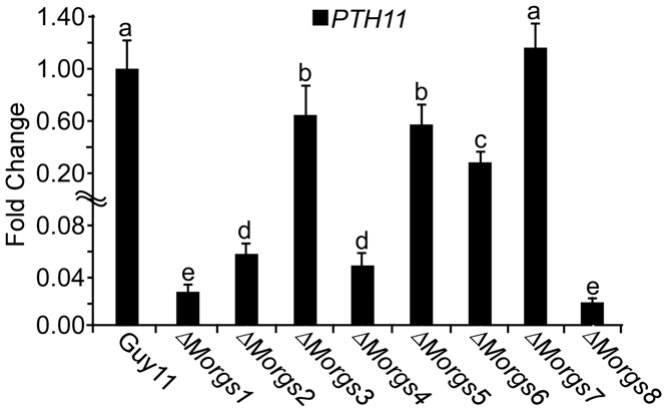
*PTH11* gene expression in Δ*Morgs* mutants. RNA was extracted from mycelia cultured in liquid CM medium at 28°C for 2 days. *ACTIN* was used for normalization, and the values were calculated by 2^-ddCT^ methods with quantitative RT-PCR data. Values represent mean ± SD from two independent experiments with three replicates each.

The pathogenicity factor MoPth11 was reported to be involved in the cAMP pathway, as its transcription is regulated by exogenous cAMP [41], [42]. Disruption of *MoPDEH* also affected the expression of *MoPTH11* during plant infection [31]. The expression of *MoPTH11* was thus assessed and found to be down-regulated in all of the mutants. The transcription of *MoPTH11* decreased more than 30-, 17-, 20-, and 20-fold, in Δ*Morgs1*, Δ*Morgs2*, Δ*Morgs4*, and Δ*Morgs8* mutants, respectively. However, in the mutants Δ*Morgs3*, Δ*Morgs5*, Δ*Morgs6*, and Δ*Morgs7*, the expression of MoPth11 was not obviously changed (Figure 6). These results indicated that the RGS proteins have differentiated roles in the transcription of *PTH11* in *M. oryzae*.

### MoRgs4 affects extracellular laccase and peroxidase activities

**Figure 7:**
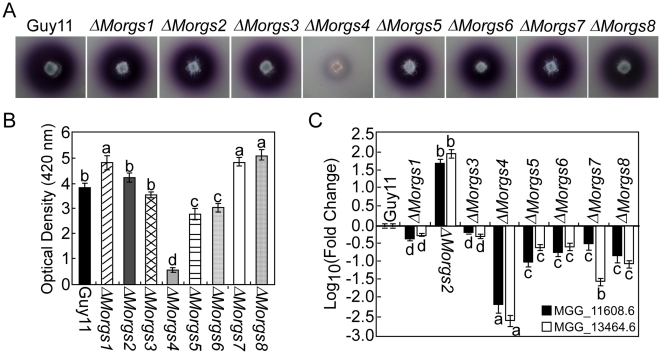
MoRgs4 has a role in the regulation of extracellular laccase activities. (A) Laccase activity was tested on CM agar medium containing 0.2 mM ABTS at final concentration. Discoloration was observed on day 2 after inoculation. (B) Laccase activity measured by ABTS oxidizing test (see Materials and Methods). (C) Quantitative RT-PCR analysis of two laccase genes in wild type and mutants. Expression data were normalized using the *ACTIN* gene. Error bars represent standard deviation. Different letters in each data column indicate significant differences at *P* = 0.01.

To determine whether any RGS proteins are involved in the regulation of laccase activity, a pathogenicity factor of certain fungi [43]-[47], we tested the mutant strains on CM agar and liquid medium supplemented with 0.2 mM 2, 2′-azino-di-3-ethylbenzthiazoline-6-sulfonate (ABTS). In each case, decreases in laccase activity were only seen in the Δ*Morgs4* mutant, with a less-oxidized dark purple stain around colonies of the mutant and a lower level of laccase activity in the culture filtrate compared with the wild-type strain (Figure 7A and 7B). Consistent with these observations, the expression levels of two extracellular laccase genes, MGG11608.6 and MGG13464.6, were also significantly down in Δ*Morgs4* mutants (Figure 7C).

**Figure 8:**
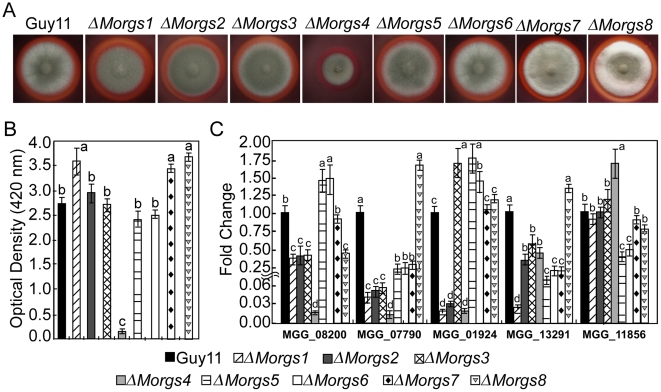
Measurement of activities of extracellular peroxidases. (A) The discoloration of Congo red was tested on the CM agar containing 200 µg/ml of the dye. Discoloration was observed on day 7 after inoculation at 28°C. (B) Peroxidase activity measured by ABTs oxidizing test under H_2_O_2_ supplemented conditions. (C) Expression profiles of five extracellular peroxidase genes in the wild type and mutant strains. Different letters in each data column indicate significant differences at *P* = 0.01.

The Congo red degradation reaction is catalyzed by peroxidase, which requires H_2_O_2_ as a limiting substrate [48], [49], [50]. Discolored halos were observed beyond the wild-type colony margins when cultured on CM agar plates with Congo red, but there was no color change with the Δ*Morgs4* mutant, implying that MoRgs4 is involved in peroxidase activity (Figure 8A). Enzyme activity assays using ABTS as substrate revealed that the Δ*Morgs4* mutant almost lost its peroxidase activity in the extracellular culture filtrate (Figure 8B). We further examined the transcriptional level of five peroxidase-encoding genes that possess a signal peptide. The expression levels of MGG08200.6, MGG07790.6, MGG_01924.6, and MGG_13291.6 were dramatically downregulated in the Δ*Morgs4* mutant and, in contrast, only MGG_11856.6 was upregulated in this mutant. A lesser degree of downregulation was observed for the other Δ*Morgs* mutants (Figure 8C). These data suggested that RGS proteins might be all involved in the regulation of extracellular peroxidases with MoRgs4 playing a more prominent role.

### MoRgs1, MoRgs3, MoRgs4, and MoRgs7 are required for full virulence

**Figure 9:**
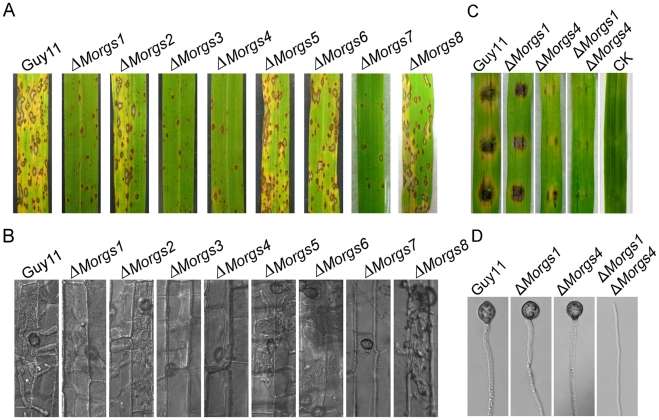
Loss of *MoRGS1*, *MoRGS3*, *MoRGS4*, and *MoRGS7* lead to a significantly reduction in pathogenicity. (A) Leaf spraying assay. Five milliliters of conidial suspension (5×10^4^ spores/ml) of each strain were sprayed on two-week old rice seedlings. Diseased leaves were photographed at 7 dpi. (B) Close observation of infectious growth. Excised rice sheath from 4-week-old rice seedlings was inoculated with conidial suspension (1×10^4^ spores/ml of each strain). Infectious growth was observed at 48 hpi. (C and D) Δ*Morgs1*Δ*Morgs4* double mutant was unable to form appressorium and completely lose pathogenicity on detached barley seedling leaves. Diseased leaves were photographed 5 days after inoculation, and hyphal plugs were incubated on hydrophobic surfaces for 48 hours allowing appressorium formation.

According to the expression profiles (Table 1), the expression of *RGS* genes, especially *MoRGS1*, *MoRGS3* and *MoRGS7*, was significant altered during infectious growth *in planta*, suggesting their potential roles in pathogenicity. To further test virulence involvement of these RGS proteins, susceptible rice seedlings of CO-39 were sprayed with conidia of various Δ*Morgs* mutants. Very few lesions were found up to 7 days post-inoculation with Δ*Morgs1*, Δ*Morgs3*, Δ*Morgs4*, and Δ*Morgs7* mutants. In contrast, rice seedlings sprayed with Δ*Morgs2*, Δ*Morgs5*, Δ*Morgs6,* and Δ*Morgs8* mutants under the same conditions developed numerous typical rice blast lesions similar to the wild type strain (Figure 9A). Since the Δ*Morgs1*, Δ*Morgs3*, Δ*Morgs4*, and Δ*Morgs7* mutants exhibited normal appressoria formation, we examined the ability of the appressoria in penetration of the onion epidermal cells. Interestingly, only Δ*Morgs3* and Δ*Morgs7* showed decreased penetration efficiency (61% and 21%) compared to the wild type (85%), while no change was found for the other Δ*Morgs* mutants (Table 2). However, the majority of the appressoria in the Δ*Morgs2*, Δ*Morgs5*, Δ*Morgs6*, and Δ*Morgs8* mutants formed invasive hyphae, while only a few limited infectious hyphae developed from appressoria of Δ*Morgs1*, Δ*Morgs3*, Δ*Morgs4*, and Δ*Morgs7* mutants (data not shown). To further validate this observation, we repeated the test with rice sheath cells and found that the appressoria of the Δ*Morgs2*, Δ*Morgs5*, Δ*Morgs6*, and Δ*Morgs8* mutants as well as the wild type strain, produced branching infectious hyphae, but the appressoria of the Δ*Morgs1*, Δ*Morgs3*, Δ*Morgs4*, and Δ*Morgs7* mutants produced only limited infectious hyphae (Figure 9B). These results indicate that MoRgs1, MoRgs3, MoRgs4, and MoRgs7 have significantly reduced virulence resulting from the defect in infectious hyphal growth.

Moreover, we examined the collective effect of MoRgs1 and MoRgs4 on pathogenicity on detached barley leaves. Consistently, Δ*Morgs1* and Δ*Morgs4* were less virulent than the wild type strain Guy11 and the Δ*Morgs1*Δ*Morgs4* double mutant strain lost all pathogenicity (Figure 9C). Further observation indicated that the Δ*Morgs1*Δ*Morgs4* was unable to form appressorium on induction surfaces (Figure 9D).

### Functions of *M. oryzae* RGS proteins in cAMP and G protein signaling

**Figure 10:**
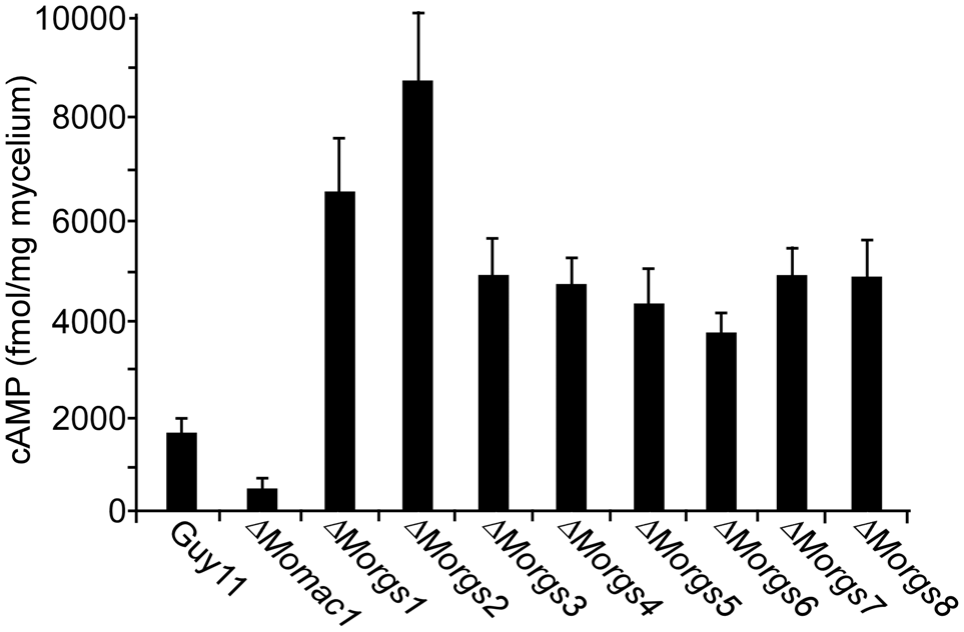
*MoRGS* genes regulate intracellular cAMP levels during pathogenesis. Loss of *MoRGS* leads to increased accumulation of total cellular cAMP levels. Bar chart showing quantification of intracellular cAMP in the mycelia of the indicated strains following 2 days of culturing in complete medium. Two biological repetitions with three replicates were assayed. The error bars represent SD of three replicates.

MoRgs1 plays an important role in regulation of the intracellular cAMP level in *M. oryzae* [28]. To determine whether other RGS and RGS-like proteins are also involved in this process, we measured intracellular cAMP levels of the mutants in the hyphal stage and compared with that of the wild-type and the Δ*Momac1* mutant strains. The results indicated that all Δ*Morgs* mutant strains accumulate somewhat higher levels of cAMP than the wild-type strain. Compared with wild-type, Δ*Morgs1* showed a ∼3.7-fold higher intracellular cAMP level, which is consistent with the earlier study [28]. Surprisingly, an increase of five-fold was found in the Δ*Morgs2* mutant while the Δ*Morgs3-8* mutants accumulated 2.8-, 2.8-, 2.2-, 2.1-, 3-, and 3-fold higher levels of cAMP, respectively (Figure 10). While the mechanism remains unclear, particularly for Δ*Morgs4* and Δ*Morgs5* mutant strains, these results suggest that RGS proteins have important roles in regulating intracellular cAMP levels.

**Figure 11:**
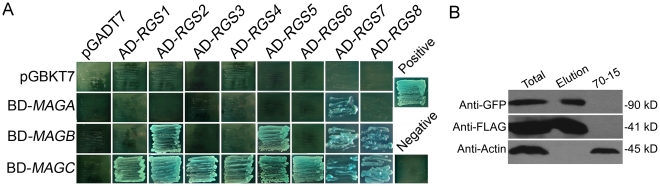
Physical interactions between RGS proteins and MoMagA, MoMagB, and MoMagC proteins. (A) Yeast transformants expressing bait (pGBKT7) and prey (pGADT7) constructs were assayed for growth on SD-Leu-Trp-His (SD-His) plates and β-galactosidase (LacZ) activities with positive and negative control. (B) co-IP assay for the interaction of MoRgs2 with MoMagB. Western blot analysis with total proteins (Total) isolated from transformants co-expressing the *MoRGS2*-GFP and *MoMAGB*-3xFLAG constructs and proteins eluted from the anti-FLAG M2 beads (Elution). The presence of MoRgs2 and MoMagB was detected with an anti-GFP and an anti-FLAG antibody, respectively. Total proteins isolated from the wild-type strain (70-15 fractions) and detection with an anti-actin antibody was included as the controls.

The RGS domain of RGS proteins has a high affinity to Gα and the binding specificity between RGS and Gα proteins often determine signal specificity and amplitude. In *S. cerevisiae*, Sst2 functions as a negative regulator of pheromones and mating by interacting with Gα Gpa1 [12] and Rgs2 down-regulates glucose activation of the cAMP pathway through direct inhibition of Gpa2 [51]. In a study by Chasse et al., Sst2, Rgs2, Rax1, and Mdm1 were all found to bind Gpa1 and affect Gpa1 signaling, although Sst2 still remained the most regulatory role in Gpa1 signaling and mating [15]. *M. oryzae* MoRgs1 was found to interact with MoMagA for pathogenicity and MoMagB for conidiation [28]. To find out whether other RGS proteins also function similarly by binding to all or specific Gα proteins, a yeast two-hybrid (Y2H) assay was conducted. In this assay, MoRgs2, MoRgs5, MoRgs7 and MoRgs8 were found to interact with MoMagB, while MoRgs7 only interacted with MoMagA. Surprisingly, all RGS and RGS-like proteins interacted with MoMagC (Figure 11A and 11B). MoRgs1 failed to interact with MoMagA in contrast to previously reported may due to the different experimental conditions utilized [28].

**Figure 12:**
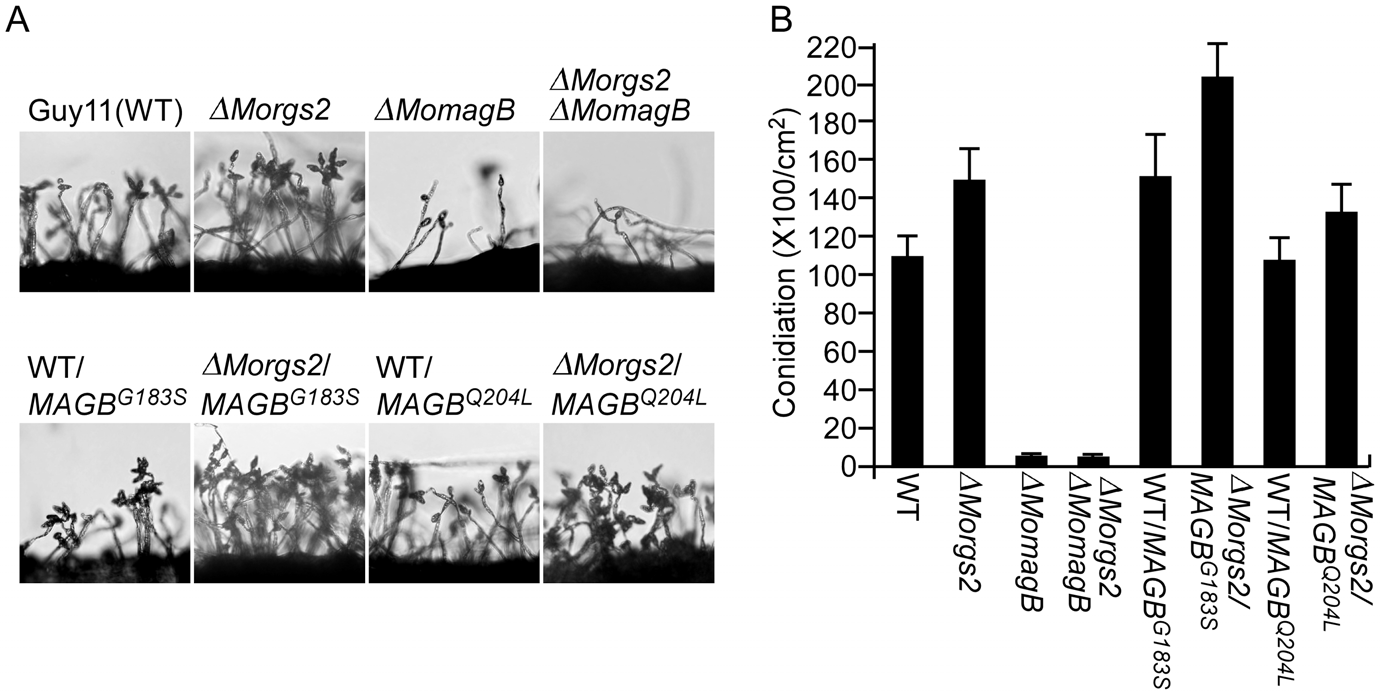
MoRgs2 regulates asexual development upstream of MoMagB. (A) Evaluation and quantification of conidiogenesis. Strains of the indicated genotypes were cultured in dark for 7 days at 28°C and then grown further for 3 days under constant illumination. Conidia and conidiophores were imaged under a microscope. (B) Conidiation defects in Δ*MoMagB* and Δ*Morgs2*Δ*MoMagB* strains. Conidia produced by the indicated strains were harvested and quantified. Data represent the mean values (±SD) from three independent experiments.

To further investigate the interaction between MoMagB and MoRgs2, a Δ*MomagB*Δ*Morgs2* double mutant strain was generated and analyzed. In addition, two RGS-insensitive mutant alleles, *MoMAGB* ^G183S^ and *MoMAGB* ^Q204L^ allele were also introduced respectively into the Δ*MomagB* mutant. Consistent with a role for MoRgs2 in conidiation that is mediated through MoMagB, no conidia was found in the Δ*MomagB*Δ*Morgs2* mutant, whereas a significant increase in conidiation was found for Δ*Morgs2* expressing *MoMAB* ^G183S^ and *MoMAGB* ^Q204L^ alleles (Figure 12).

These results indicated that MoRgs2 functions upstream of MoMagB in conidiation. MoRgs7 regulates pathogenicity may also be mediated through MoMagB, similar to MoRgs1. The interactions between MoRgs5 and MoRgs8 and MoMagB could confer additional roles in controlling various developmental processes.

## Discussion

Heterotrimeric G-proteins play crucial roles in the regulation of fungal developmental processes and pathogenicity [7]. RGS proteins function as negative regulators to enhance the intrinsic GTPase activity of Gα subunits, thereby inactivating the G-proteins and rapidly switching off the cellular response. A large number of mammalian RGS proteins have been shown to play important roles in various signaling pathways [52]. Many fungal RGS protein homologs have also been well characterized, such as Sst2 of *S. cerevisiae* [12], FlbA and RgsA of *Aspergillus nidulans* [53]–[55], CPRGS-1 of *Cryphonectria parasitica* [56], and Cag8 of *Metarhizium anisopliae* [57], as well as MoRgs1 of *M. oryzae* [28]. Here, we identified and characterized seven RGS proteins in addition to MoRgs1 of *M. oryzae*. Our findings revealed that there are as many as eight RGS and RGS-like proteins in *M. oryzae*, the most ever found in fungi, which play various roles in the modulation of vegetative growth, asexual/sexual development, cell wall integrity, surface hydrophobicity, appressorium formation and penetration, and pathogenicity in this pathogenic fungus (Table S2).

The *M. oryzae* MoRgs1 is highly homologous to *A. nidulans* FlbA and *C. parasitica* CPRGS-1. Consistent with studies of FlbA, and CPRGS-1 that positively regulate asexual development [54], [56], our data indicated that MoRgs1 positively regulates asexual sporulation. This, however, contradicted with the previous report that MoRgs1 has a negatively role in asexual development reported. Regardless, our findings for the role of MoRgs1 are mostly consistent with the previous study [28]. In *M. oryzae*, deletion of the gene encoding MoMagA or MoMagC has no effect on vegetative growth, appressorium formation, or pathogenicity, except that deletion of *MoMAGC* encoding MoMagC exhibited reduced conidiation [19]. The Δ*MomagB* mutant, however, exhibited significantly reduced vegetative growth, conidiation, and appressorium formation, as well as virulence [19]. A reduction in perithecium formation was observed in Δ*MomagA,* but not Δ*MomagC*, and no perithecium was found in Δ*MomagB* (Figure S3). The strains with the putative dominant active *MoMAGB* ^G42R^ allele formed appressoria on both hydrophobic and hydrophilic surfaces [26]. This phenotype was also observed in the transformant carrying multiple copies of *MoMGB1* [24] and in a phosphodiesterase gene mutant Δ*MopdeH* [31], similar to results observed in the Δ*Morgs1* mutant, suggesting that expression of *MoMAGB* ^G42R^ and multiple copies of *MoMGB1*, inactivation of *MoPDEH*, and deletion of *MoRGS1* all enhance cAMP signaling. Because of this, it is feasible to hypothesize that MoRgs1 has a negative effect on Gα MoMagB signaling. The *MoMAGB* ^G42R^ mutation also reduced conidiation and virulence, failed to form asci, and caused autolysis of aged colonies [26], and the same phenotypes appeared in the Δ*Morgs1* mutant, suggesting that MoRgs1 negatively regulates MoMagB. Surprisingly, MoRgs1 and MoMagB failed to interact with each other in our hand.

Instead, interactions between MoRgs7 and MoMagA, MoRgs2, MoRgs5, MoRgs7, MoRgs8 and MoMagB, and MoRgs1-8 and MoMagC were found. These interactions could suggest functional significance (differentiated interactions with MoMagA and MoMagB) or not (interactions with MoMagC). Indeed, MoRgs1 and MoRgs4 likely positively regulate MoMagB in conidiation, whereas MoRgs2 and MoRgs3 negatively regulate MoMagB and or MoMagC in this process. For sexual reproduction, MoRgs4 may be involved in a similar mechanism to MoRgs1, but extra and different regulators must exist in the regulation of MoRgs1 and MoRgs4, since the Δ*Morgs4* mutant produced few asci while the Δ*Morgs1* mutant produced no asci. Moreover, MoRgs2, MoRgs5, MoRgs7 and MoRgs8 were found to interact with MoMagB. While no phenotypic changes exhibited by MoRgs5, the Δ*Morgs2* mutant did show increased conidiation, suggesting that MoRgs2 may negatively regulate MoMagB in conidiogenesis and pathogenesis. Since MoRgs4 and MoRgs5 are structurally more similar to *S. cerevisiae* Mdm1 whose role in G protein was not established, their roles in *M. oryzae* may be established independent of G protein signaling.

Interestingly, with the exception of Δ*Morgs5* and Δ*Morgs8*, most Δ*Morgs* mutants often formed multiple appressoria on hydrophobic surfaces, which were also observed in the site-directed mutation transformant *MoMGB1* ^D41N^, as the aspartic acid residue at 41 is known to be involved in the interaction between MoMgb1 and MgSte20 [24]. This may indicate that RGS proteins could play a role in activating the D41 of MoMgb1 or have cross-talk between MoSte20 (MgSte20) signaling for germ tube growth and correct regulation of appressorium formation.

The fungal cell wall plays important roles during cell division, growth, and morphogenesis, and in mediating all exchanges between the cell and its environment [58], [59]. In pathogenic fungi, the ability to maintain cell wall integrity is critical to the establishment of disease in the host [33]. Several cell wall integrity-associated genes such as MoMps1 and MoMck1 have been characterized in *M. oryzae.* MoMps1 and MoMck1 have been described as essential for cell wall integrity and pathogenicity [32], [33]. In our latest studies, a constitutive activating cAMP pathway mutant Δ*MopdeH* also showed an autolysis phenotype like that observed in the Δ*Morgs1* mutant [31]. Thus, the G-protein/cAMP signaling pathway may have cross-talk with the MAPK pathway in regulating cell wall integrity. Alternatively, like the Δ*MopdeH* mutant, the cell wall integrity defect in Δ*Morgs1* could be due to the high intracellular cAMP level in the mutant. However, based on our results, there should be more regulators involved in regulation of cell wall integrity, because other Δ*Morgs* mutants besides Δ*Morgs1* also have high intracellular cAMP levels but do not exhibit the autolysis phenotype.

Most hydrophobins confer surface hydrophobicity on fungi forming a spore rodlet layer. Deletion of several hydrophobin genes, including *MoMPG1*, resulted in a water- or detergent-soaked, easily wettable phenotype and these genes played important roles in multiple infection-related processes [34]–[39]. In previous studies, G-protein and cAMP signaling pathways have been reported to be involved in hydrophobin synthesis and surface hydrophobicity [31], [57]. In the insect pathogenic fungus *M. anisopliae*, loss of the *MoRGS1* homolog gene *Mocag8* reduced the transcription of a hydrophobin-encoding gene [57]. In the chestnut blight fungus *C. parasitica*, the RGS protein CPRGS-1 is also known to regulate hydrophobin synthesis [56]. In *M. oryzae*, a constitutive activate cAMP signaling mutant Δ*MopdeH* showed a defect in surface hydrophobicity and a low level of *MoMPG1* expression [31]. These results well support the surface hydrophobicity defects of the Δ*Morgs1* and Δ*Morgs4* mutants. Furthermore, they can also be taken as evidence explaining the cell wall integrity defect in Δ*Morgs1*.

The high expression level of *RGS* genes at late infection stages in infected rice leaves indicates their potential role in infectious growth and virulence. Mutation of *MoRGS1*, *MoRGS3*, *MoRGS4*, and *MoRGS*7 significantly reduced virulence and produced fewer lesions than the wild-type strain, implying that appressoria formed by these mutants are probably defective in penetration. It is likely that these four genes regulate processes involved in the early stages of appressorium penetration, such as development of the penetration peg or differentiation of infectious hyphae. The reduction in pathogenicity may be due to a reduction in development at the pre-penetration stages or a defect in infectious growth of Δ*Morgs* mutants in host cells. According to the penetration data, *MoRGS1*, *MoRGS3* and *MoRGS7* are indeed involved in penetration and infectious growth and well support the hypothesis. However, unlike Δ*Morgs1*, Δ*Morgs3* and *MoRGS7*, the reduced virulence of Δ*Morgs4* mainly resulted from the defect in infectious hyphal growth, indicating the unique functions of each RGS protein in *M. oryzae*. RGS proteins may act as different regulators in the regulation of different targets (such as Gα subunits in pathogenesis).

In the present study, Δ*Morgs4* totally lost laccase and peroxidase activity. Laccases are copper-containing oxidases found in many plants, fungi, and microorganisms. Laccase activity has been reported to be involved in virulence in some fungi [43]. In *M. oryzae*, several virulence attenuation mutants also show loss or reduction of laccase and peroxidase activities [44], [45], [60]. Therefore, the loss of laccase and extracellular peroxidase activity might be one aspect of the reduced virulence in the Δ*Morgs4* mutant. However, Δ*Morgs1* and Δ*Morgs3* also had reduced pathogenicity but with normal laccase and extracellular peroxidase activity, indicating regulators other than laccases and extracellular peroxidases must exist in the G-protein signaling pathway to control infection-related processes in *M. oryzae*. The disparity may also indicate that the function of MoRgs4 may be more in line with that of *S. cerevisiae* Mdm1 and could be independent of G protein signaling.

Overall, our results indicate that different RGS proteins control unique signal transduction pathways in *M. oryzae*, which are involved in asexual/sexual development, appressorium differentiation, penetration, and infectious growth. It will be important and interesting to distinguish specific functions associated with each RGS proteins and link G-protein signaling to the pathogenicity of the fungus.

## Materials and Methods

### Strains and culture conditions

The *M. oryzae* Guy11 and 70–15 strains were used as wild type for transformation in this study. All strains were cultured on complete medium (CM) agar plates [31]. Liquid CM medium was used to prepare the mycelia for DNA and RNA extraction. For conidiation, strain blocks were maintained on straw decoction and corn (SDC) agar media [31] at 28°C for 7 days in the dark followed by 3 days of continuous illumination under fluorescent light.

### Targeted gene deletion and complementation

Standard DNA and RNA manipulations were performed as described previously [61]. The gene-deletion mutants were generated using the standard one-step gene replacement strategy. First, two 1.0 kb of sequences flanking of targeted gene were PCR amplified with primer pairs (Table S1), then a ∼2-kb fragment containing the two flanking sequences was amplified by overlap PCR. All amplified sequences and fragments were sequenced and then ligated to flank the hygromycin resistance cassette, which was amplified with primers FL1111 & FL1112 (Table S1), into the pMD19-T vector (Takara Co. Dalian, China). The ∼3.4-kb fragments, which contain the flanking sequences and hygromycin cassette, were amplified and transformed into protoplasts of wild type Guy11. The complement fragments, which contain the entire *RGS* genes and their native promoter regions, were amplified by PCR with primers (Table S1) and inserted into pCB1532 (sulphonylurea resistance) or pYF11 (bleomycin resistance) to complement the mutant strains, respectively.

### Vegetative growth

Small agar blocks were cut from the edge of 4-day-old cultures and placed onto CM and SDC media for culturing in the dark at 28°C. The size and morphology of the colonies were examined each day for 10 days and then photographed. The experiment was performed in triplicate.

### Hyphal growth and surface hydrophobicity assay

For hyphal growth, small agar blocks were cut from the edge of 4-day-old cultures and placed onto the CM and CM adding 1 M sorbitol and cultured in the dark at 28°C for two weeks. The size and morphology of the colonies were examined every day and photographed on day 14 after incubation. For surface hydrophobicity assay, the strains were plated onto CM agar plates and incubated at 28°C for 14-day. Sterile distilled water (10 µl) was placed on the surface of cultures. In addition, wettability of aerial hyphae to solution containing both 0.02% SDS and 5 mM EDTA was also assessed as previously described [62].

### Appressorium formation, cuticle penetration, and infection assays

Conidia were harvested from 10-day-old cultures, filtered through three layers of lens paper, and resuspended to a concentration of 5×10^4^ spores per milliliter in sterile water. For appressorium formation and cuticle penetration assays, droplets (30 µl) of conidial suspension were placed on plastic cover slips (hydrophobic), Gelbond films (hydrophilic) and onion epidermal cells and incubated under humid conditions at room temperature as described previously [63]. Appressorium formation and development of invasive hyphae were examined after incubation for 24 hours. For plant infection assays, conidia were resuspended to a concentration of 5×10^4^ spores per milliliter in a 0.2% (w/v) gelatin solution. Two-week-old seedlings of rice (*Oryza sativa* cv CO39) were sprayed with 5 ml of conidial suspension of each treatment. Inoculated plants were kept in a growth chamber at 25°C with 90% humidity and in the dark for the first 24 hours, followed by a 12/12 hours light/dark cycle [64]. Lesion formation was observed daily and photographed 7 days after inoculation.

For microscopic observation of penetration and infectious hyphae expansion in rice tissue, rice cultivar CO-39 were prepared as previously described [44] and inoculated with 100 µl of conidial suspension (1×10^4^ spores per milliliter) on the inner leaf sheath cuticle cells. After 48 hours incubation under humid conditions at room temperature, the leaf sheaths were observed under a microscope. Appressorium turgor was measured by incipient cytorrhysis (cell collapse) assay using a 1–5 molar concentration of glycerol solution as described previously [65].

### Mating

Plugs of Δ*Morgs* mutants and control strain Guy11 (*MAT1-2*) and the mating partner strain TH3 (*MAT1-1*) were point-inoculated 3 cm apart on oatmeal agar medium and incubated at 20°C under constant fluorescent light for 3 to 4 weeks. Mature perithecia were crushed to examine the asci and ascospores approximately 20 to 25 days post-inoculation.

### Intracellular cAMP, laccase and peroxidase activities assays

Two-day-old liquid mycelial cultures were harvested, frozen in liquid nitrogen and lyophilized for 16 hours. Intracellular cAMP extraction was followed as previously described [28]. The cAMP levels were quantified according to the cAMP Biotrak Immuno-assay System (BD Bioscience, NJ, USA).

Laccase and peroxidase activities were measured from 2-day-old CM liquid cultures. Mycelia were removed completely by filtration and centrifugation (5,000 g at 4°C) and processed using a colorimetric determination as described previously [66].

### Yeast two-hybrid assay

The bait constructs were generated by cloning *MoMAGA*, *MoMAGB* and *MoMAGC* full-length cDNAs into pGBKT7, respectively. The *RGS* cDNAs (*MoRGS1*, *MoRGS4*, *MoRGS5*, *MoRGS6*, *MoRGS7* and *MoRGS8*: *RGS* domain only; *MoRGS2* and *MoRGS3*: full-length) were cloned into pGADT7 as the prey constructs (see primers in Table S1). The resulting prey and bait constructs were confirmed by sequencing analysis and transformed in pairs into yeast strain AH109 as the description of BD library construction & screening kit (Clontech, USA). The Trp+ and Leu+ transformants were isolated and assayed for growth on SD-Trp-Leu-His-Ade medium and the expression of LacZ reporter gene following the instructions provided by Clontech. Yeast stains for positive and negative controls were from the Kit.

### Co-immunoprecipitation (co-IP) and western blot analysis

The *MoMAGB*-3xFLAG and *MoRGS2*-GFP constructs were generated with the yeast gap repair approach [23], [67] and confirmed by sequencing analysis. The resulting fusion constructs were co-transformed into protoplasts of 70-15. Transformants expressing the *MoMAGB*-3xFLAG and *MoRGS2*-GFP constructs were identified by PCR and confirmed by western blot analysis with an anti-FLAG antibody (Sigma-Aldrich, USA). For co-IP assays, total proteins were isolated from vegetative hyphae as described [23] and incubated with anti-FLAG M2 beads (Sigma-Aldrich). Western blots of proteins eluted from the M2 beads were detected with the anti-GFP [23], anti-FLAG and anti-actin (Sigma-Aldrich) antibodies with the ECL Supersignal System (Pierce, USA).

### Construction of the *MoMAGB^G183S^* and *MoMAGB^Q204L^* alleles

PCR products containing the native promoter of *MoMAGB* were amplified with primers FL9963/FL9965 (Table S1) and co-transformed with fragments amplified with primers FL9966/FL9964 (Table S1) into the yeast strain XK1-25 with *Xho*I digested vector pYF11 that contains the bleomycin-resistant gene and the GFP gene [23]. Plasmid pYF11::*MoMAGB^G183S^* was rescued from the resulting Trp^+^ yeast transformants. The same strategy was used to generate the pYF11::*MoMAGB^Q204L^* vector (PCR products amplified with primers FL9963/FL9967 and FL9968/FL9964, respectively, Table S1). Protoplasts of the Guy11 and Δ*Morgs2* mutant were transformed with pYF11::*MoMAGB^G183S^* or pYF11::*MoMAGB^Q204L^*.

## Supporting Information

Figure S1 **Schematic representation and verification by Southern hybridization and PCR of** ***MoRGS*** **gene disruption.** (A) Strategy of knocking out *MoRGS* genes in *M. oryzae* genome. Thick arrows indicate orientations of the *MoRGS* and hygromycin phosphotransferase (*hph)* genes. Thin lines below the arrows indicate the probe sequence of each gene. (B) Southern blot analyses of *MoRGS* gene knockout mutants with gene specific probe (probe1). Genomic DNAs of the wild-type strain and the knockout mutants were digested with corresponding restriction enzymes. The restriction enzymes are *Hind*III (HD), *Eco*RV (EV), *EcoR*I (EI), *Xba*I (XI), *Kpn*I (KI) and *Cla*I (CI). (C) RT-PCR analyses of *MoRGS* gene knockout mutants. Total RNAs of the wild-type strain and the knockout mutants were isolated and the expression levels of target gene were detected using *ACTIN* as control. No transcripts were detected in the mutants. (D) Southern blot analyses of *MoRGS* gene knockout mutants with *hph* probe (probe2).
(DOCX)

Figure S2 **Confirmation of target gene replacement.** (A) Verification mutants by PCR with one primer from resistant gene (hygromycin/ bleomycin- resistant) and one primer beyond gene flanking sequence. M, 2000 bp plus marker; g, genomic DNA; -, negative control. (B) Mutants further confirmed by qRT-PCR. Δ*Morgs1*Δ*Morgs4* double mutant was obtained by deletion *MoRGS1* in Δ*Morgs4* background.
(TIF)

Figure S3 ***M. oryzae*** **Gα subunits MoMagA and MoMagB are involved in sexual reproduction.** Perithecia development by wild type and Gá mutant strains were photographed three weeks after inoculation. Cross between TH3 (*MAT1-1*) and Guy11 (*MAT1-2*) represents the positive control. Cross of Δ*MomagA* with TH3 produced less peritheria. While Δ*MomagB* cross with TH3 failed to form peritheria. Arrow indicates the peritheria.
(TIF)

Table S1 **Primers used in this study.**
(DOC)

Table S2 **Gene deletion mutant phenotype comparison with wild type Guy11.**
(DOC)
