# Correction: Eight RGS and RGS-like Proteins Orchestrate Growth, Differentiation, and Pathogenicity of *Magnaporthe oryzae*

**DOI:** 10.1371/journal.ppat.1008187

**Published:** 2019-11-15

**Authors:** Haifeng Zhang, Wei Tang, Kaiyue Liu, Qian Huang, Xin Zhang, Xia Yan, Yue Chen, Jiansheng Wang, Zhongqiang Qi, Zhengyi Wang, Xiaobo Zheng, Ping Wang, Zhengguang Zhang

The authors would like to correct Figs 7, 8 and 9. In these three figs, errors were introduced during the preparation of figures for publication. Duplicate images are mistakenly illustrated in Figs 7A, 8A and 9A, respectively. The authors have now repeated the experiments and provided the new images. The authors confirm that these changes do not alter any findings.

**Fig 7:**
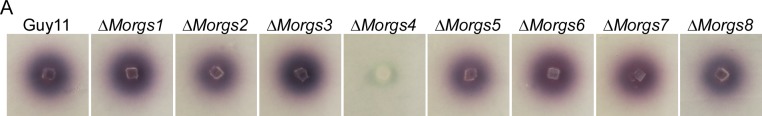
MoRgs4 has a role in the regulation of extracellular laccase activities. (A) Laccase activity was tested on CM agar medium containing 0.2 mM ABTS at final concentration. Discoloration was observed on day 2 after inoculation.

**Fig 8:**
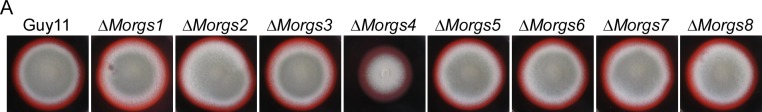
Measurement of activities of extracellular peroxidases. (A) The discoloration of Congo red was tested on the CM agar containing 200 mg/ml of the dye. Discoloration was observed on day 7 after inoculation at 28°C.

**Fig 9:**
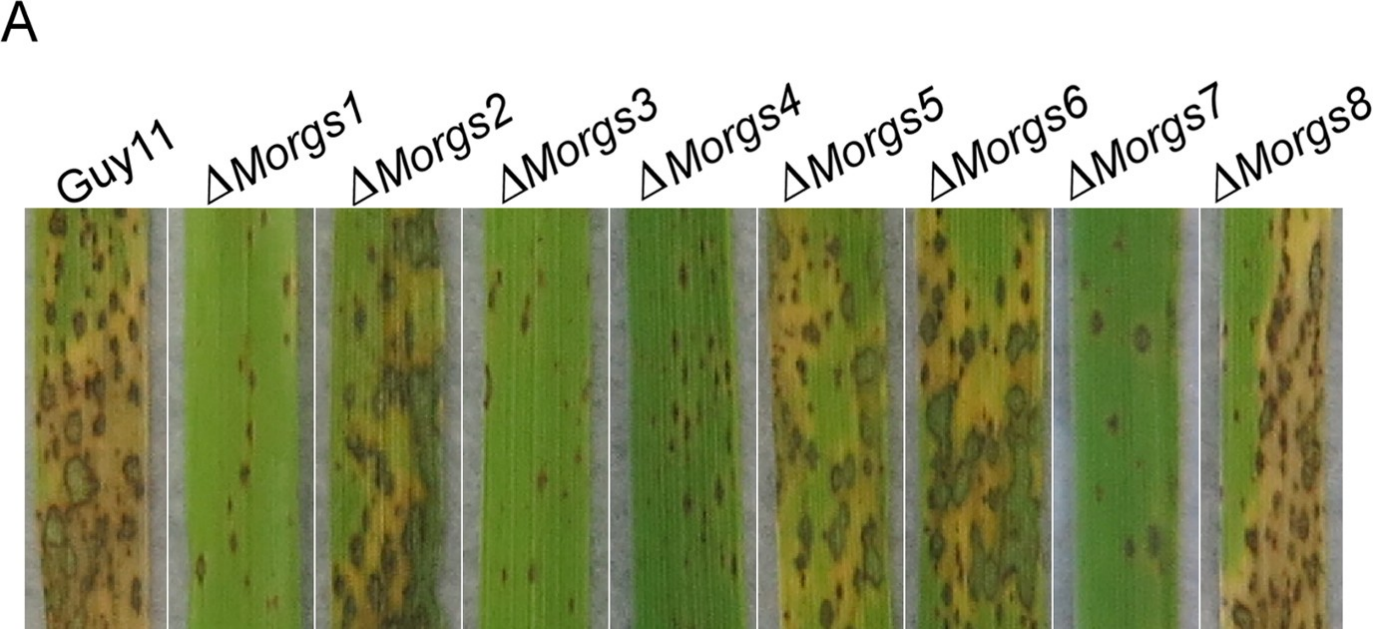
Loss of *MoRGS1*, *MoRGS3*, *MoRGS4*, and *MoRGS7* lead to a significantly reduction in pathogenicity. (A) Leaf spraying assay. Five milliliters of conidial suspension (5×10^4^ spores/ml) of each strain were sprayed on two-week old rice seedlings. Diseased leaves were photographed at 7 dpi.
