# Supplementary material for: Eight RGS and RGS-like Proteins Orchestrate Growth, Differentiation, and Pathogenicity of Magnaporthe oryzae
Source: PLoS Pathog. 2011 Dec 29;7(12):e1002450. doi: 10.1371/journal.ppat.1002450 (PMC3248559; doi:10.1371/journal.ppat.1002450)
Supplement: Figure S1 — Schematic representation and verification by Southern hybridization and PCR of MoRGS gene disruption. (A) Strategy of knocking out MoRGS genes in M. oryzae genome. Thick arrows indicate orientations of the MoRGS and hygromycin phosphotransferase (hph) genes. Thin lines below the arrows indicate the probe sequence of each gene. (B) Southern blot analyses of MoRGS gene knockout mutants with gene specific probe (probe1). Genomic DNAs of the wild-type strain and the knockout mutants were digested with corresponding restriction enzymes. The restriction enzymes are HindIII (HD), EcoRV (EV), EcoRI (EI), XbaI (XI), KpnI (KI) and ClaI (CI). (C) RT-PCR analyses of MoRGS gene knockout mutants. Total RNAs of the wild-type strain and the knockout mutants were isolated and the expression levels of target gene were detected using ACTIN as control. No transcripts were detected in the mutants. (D) Southern blot analyses of MoRGS gene knockout mutants with hph probe (probe2). (DOCX) [file ppat.1002450.s001.docx]

***MoRGS1***


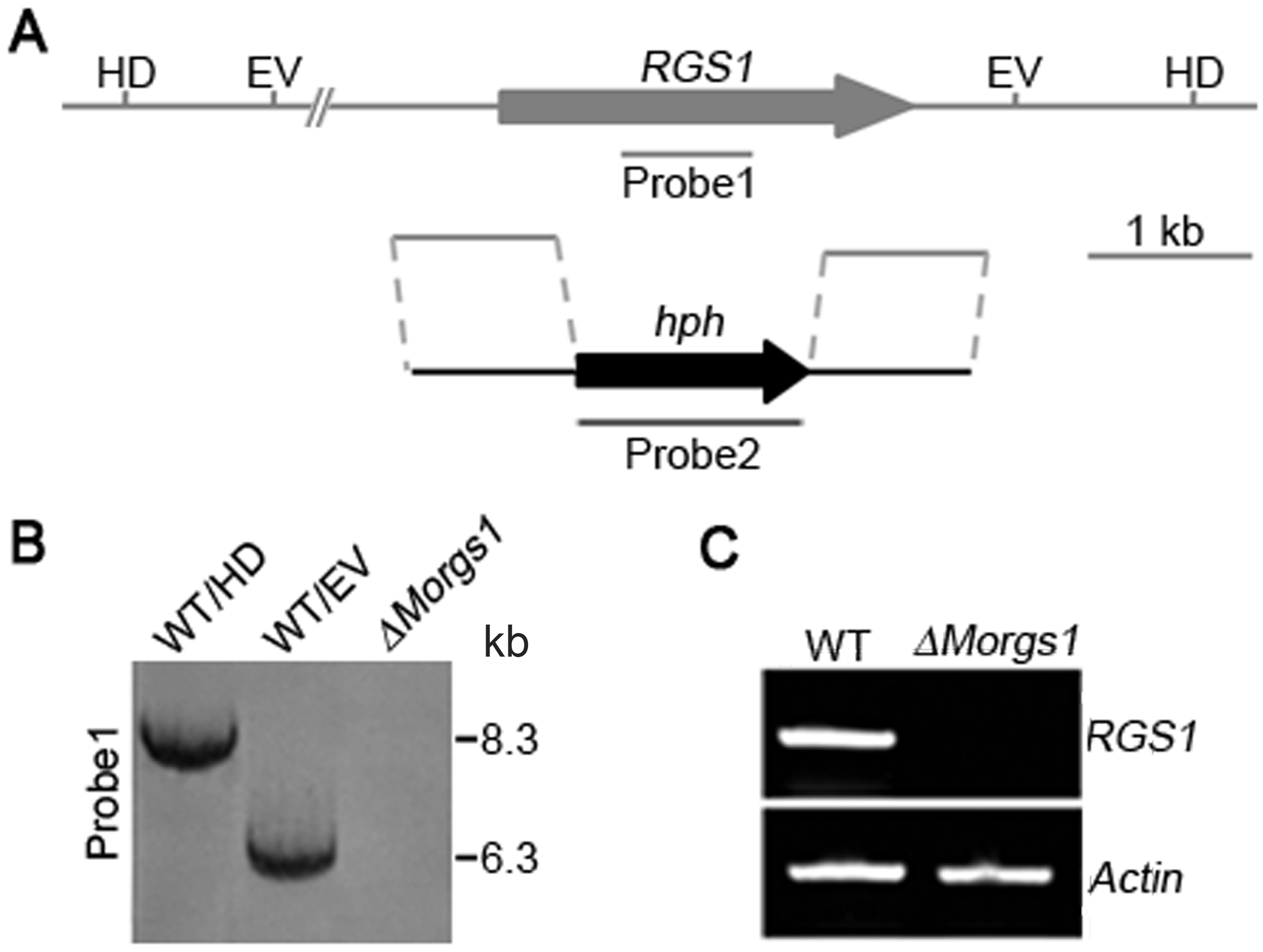


***MoRGS2***


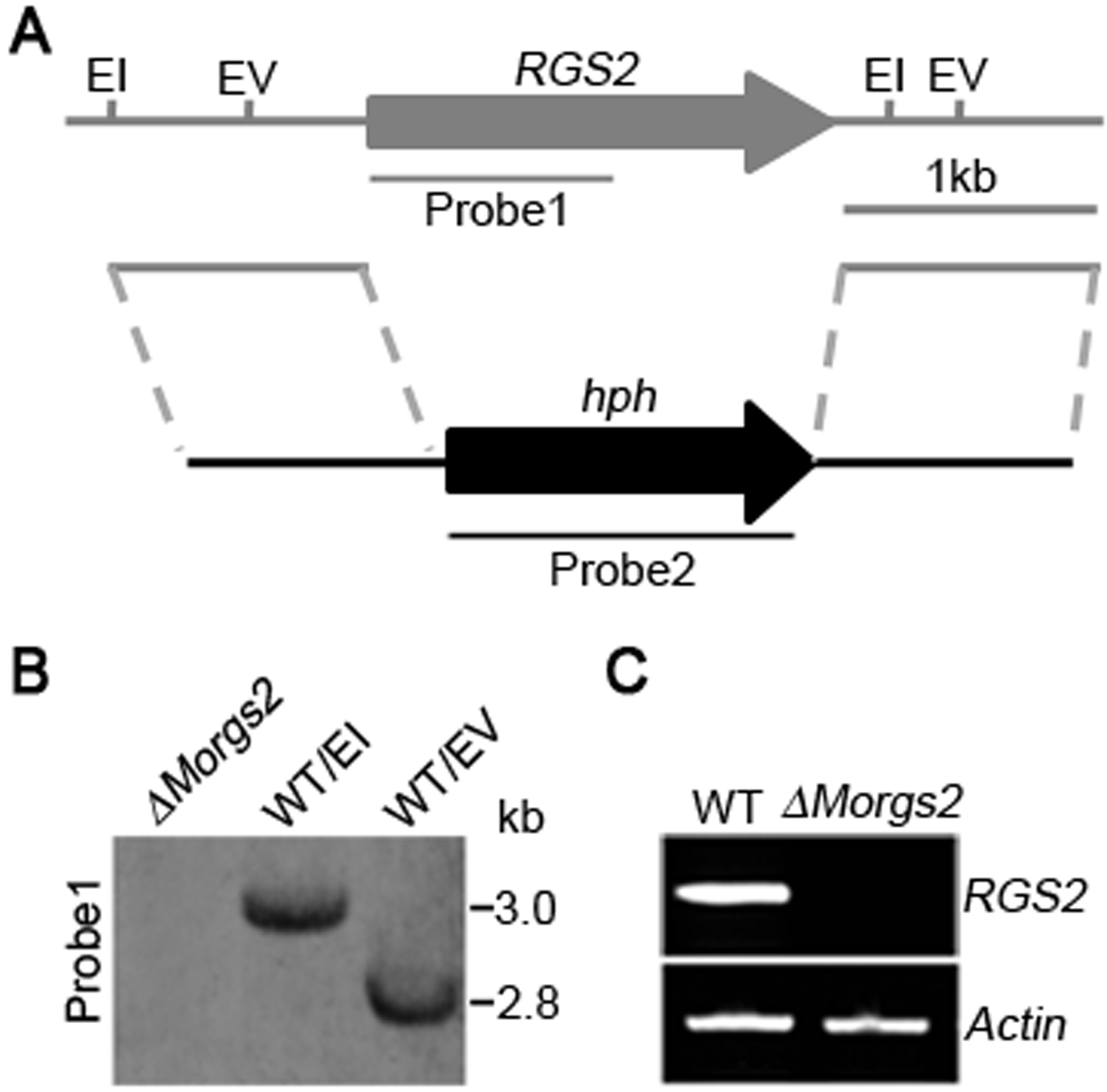


***MoRGS3***


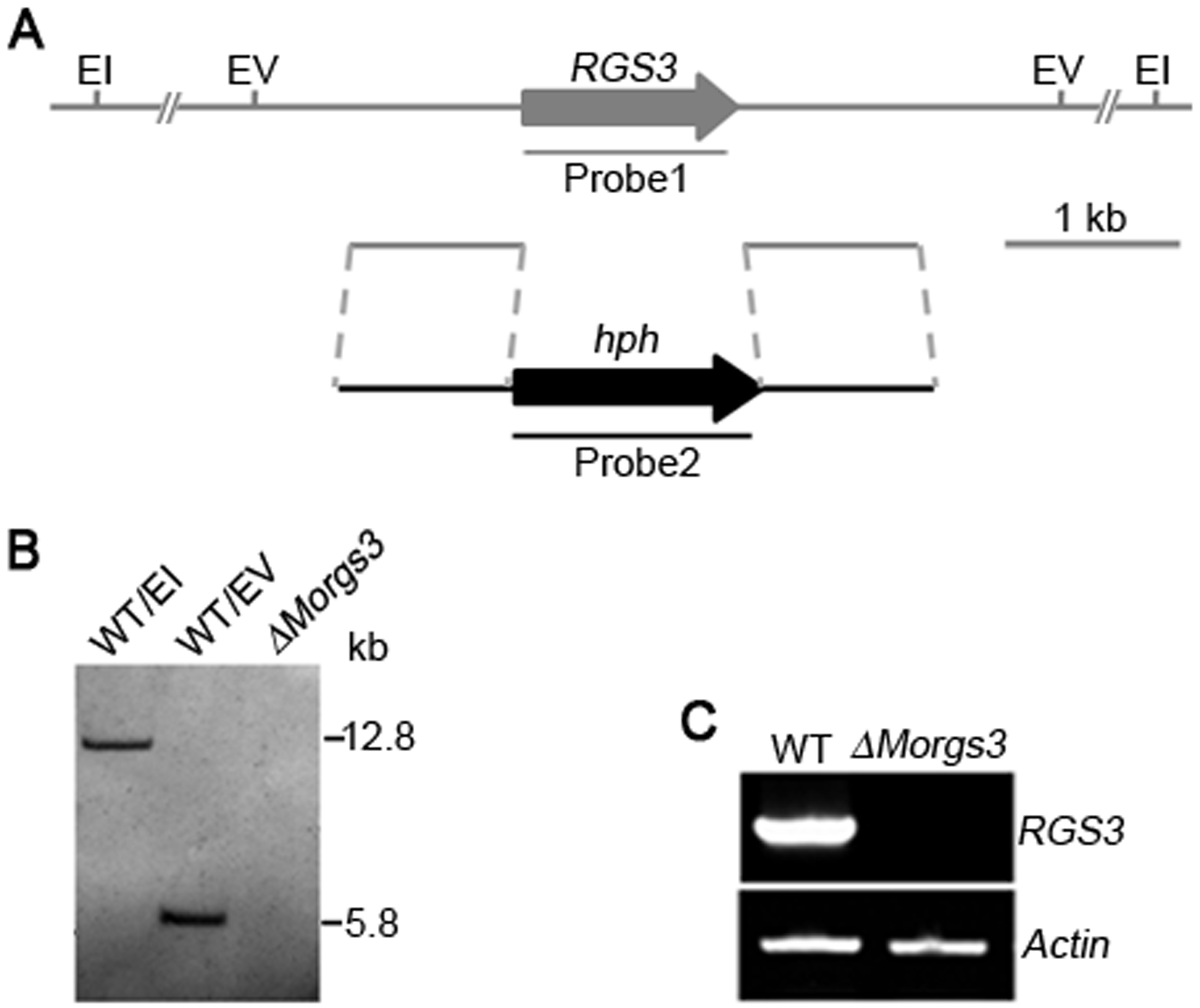


***MoRGS4***


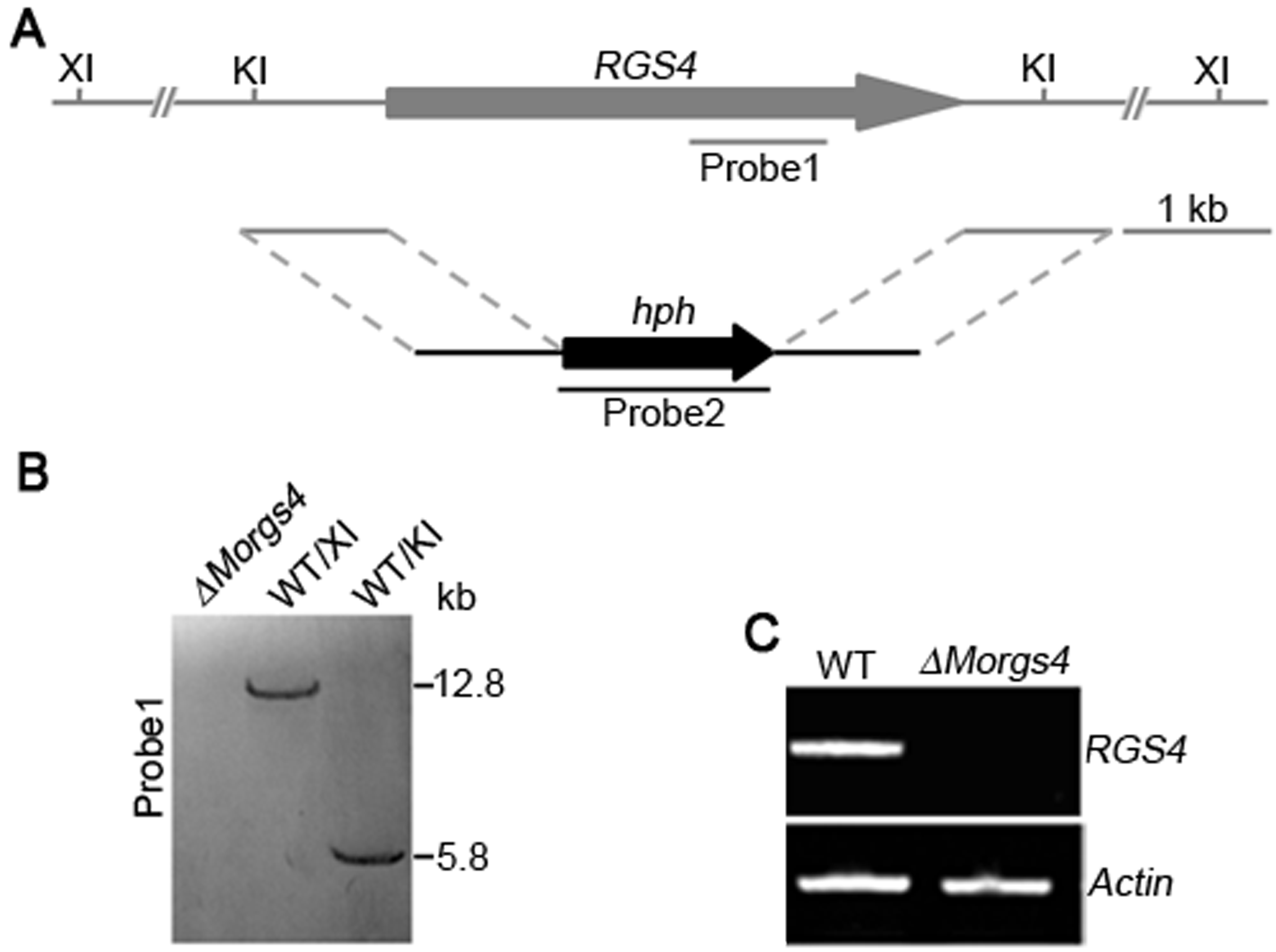


***MoRGS5***


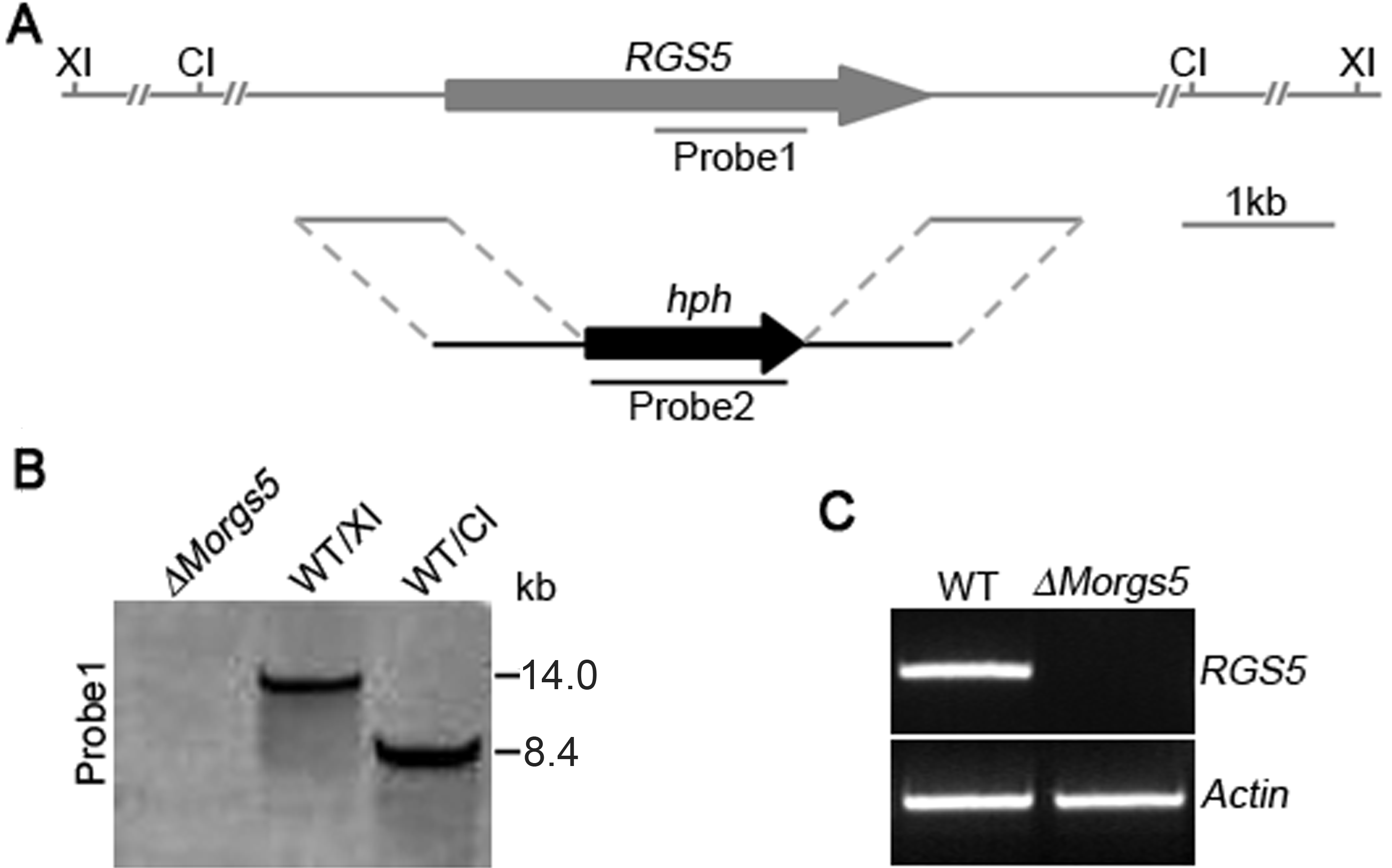


***MoRGS6***


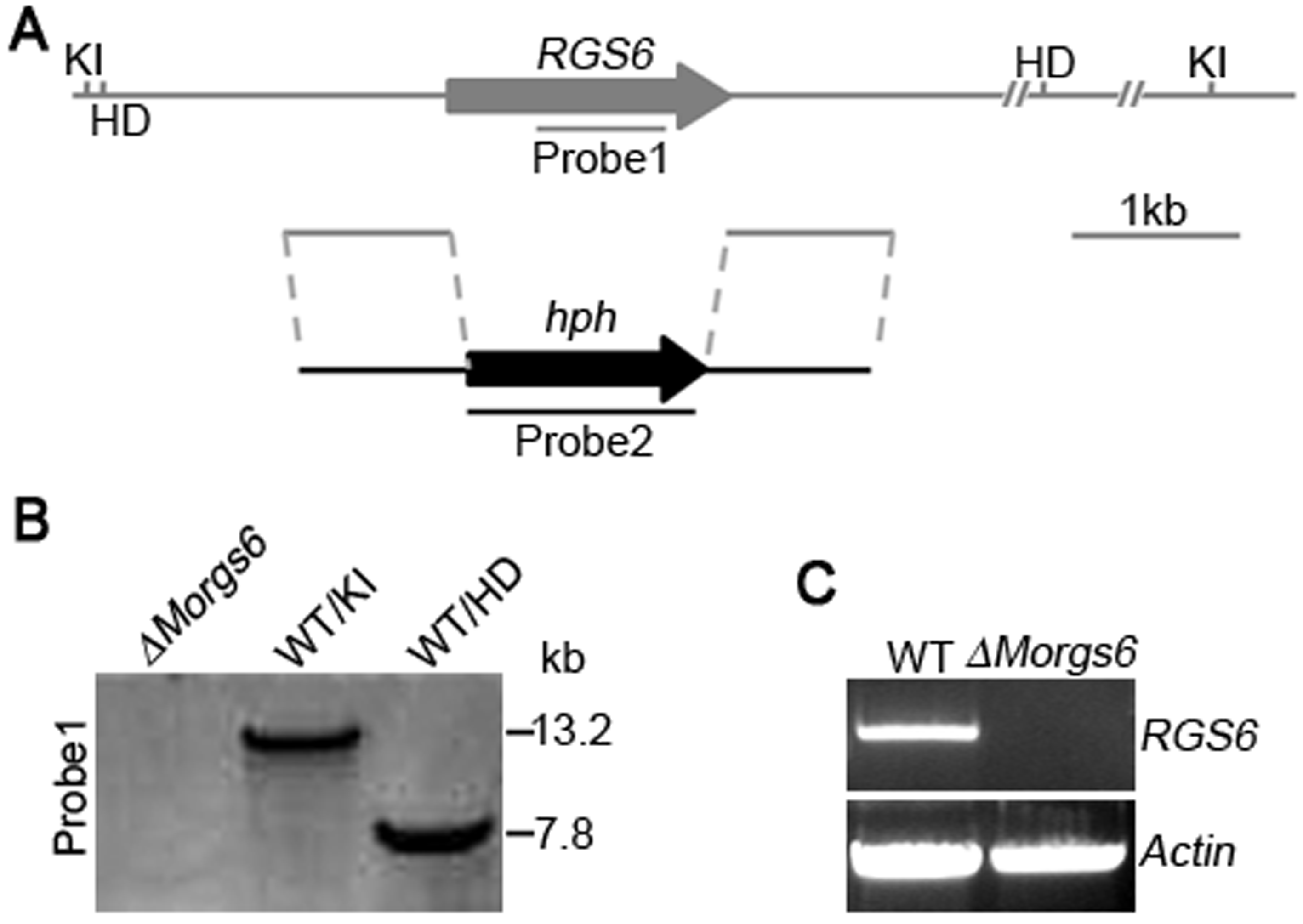


***MoRGS7***


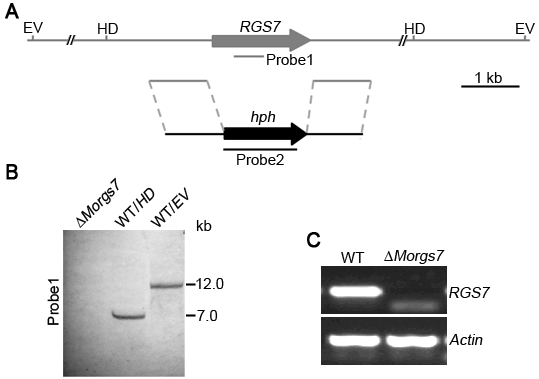


***MoRGS8***


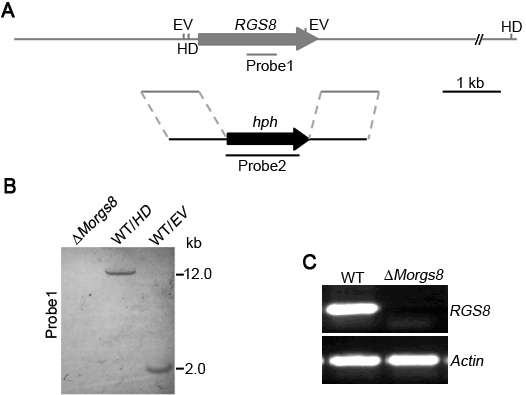

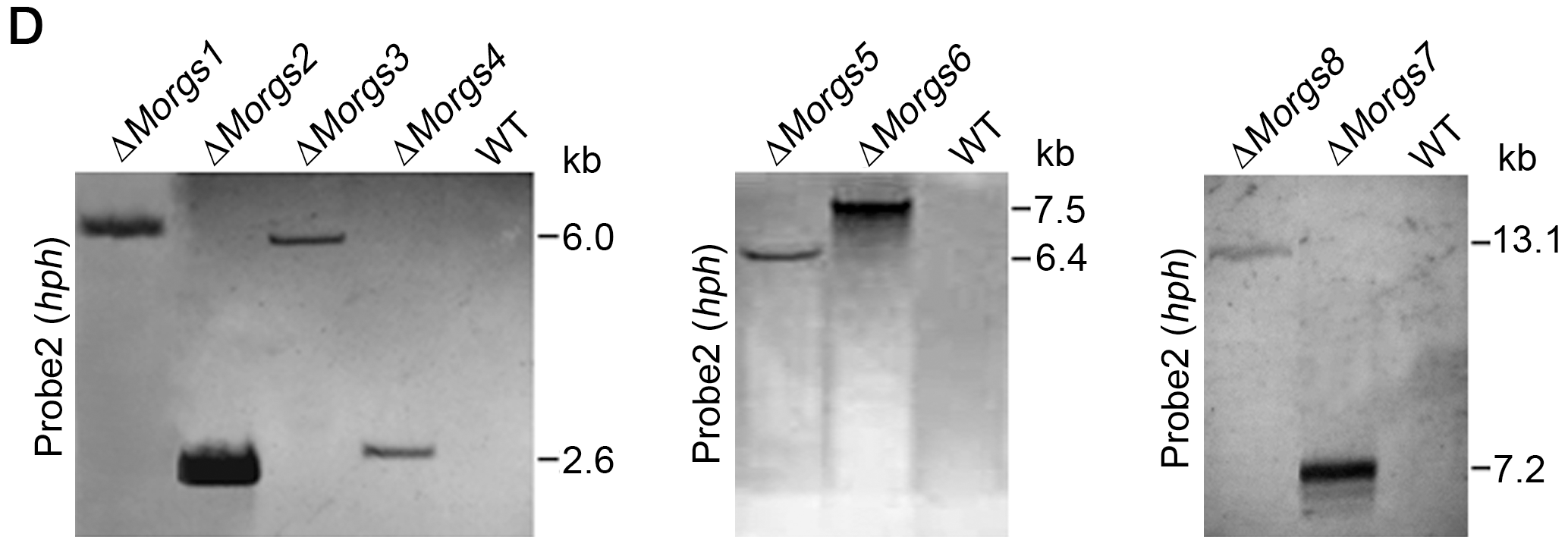


**Figure S1.** **Generation of eight *MoRGS* gene deletion mutants.** (A) Strategy of knocking out *RGS* genes in *M. oryzae* genome. Thick arrows indicate orientations of the *RGS* and hygromycin phosphotransferase (*hph)* genes. Thin lines below the arrows indicate the probe sequence of each gene. (B) Southern blot analyses of *RGS* gene knockout mutants with gene specific probe (probe1). Genomic DNAs of the wild-type strain and the knockout mutants were digested with corresponding restriction enzymes. The restriction enzymes are *Hind*III (HD), *Eco*RV (EV), *EcoR*I (EI), *Xba*I (XI), *Kpn*I (KI) and *Cla*I (CI). (C) RT-PCR analyses of *RGS* gene knockout mutants. Total RNAs of the wild-type strain and the knockout mutants were isolated and the expression levels of target gene were detected using *ACTIN* as control. No transcripts were detected in the mutants. (D) Southern blot analyses of *RGS* gene knockout mutants with *hph* probe (probe2).
