# Supplementary figures and images for: Eight RGS and RGS-like Proteins Orchestrate Growth, Differentiation, and Pathogenicity of Magnaporthe oryzae
Source: PLoS Pathog. 2011 Dec 29;7(12):e1002450. doi: 10.1371/journal.ppat.1002450 (PMC3248559; doi:10.1371/journal.ppat.1002450)

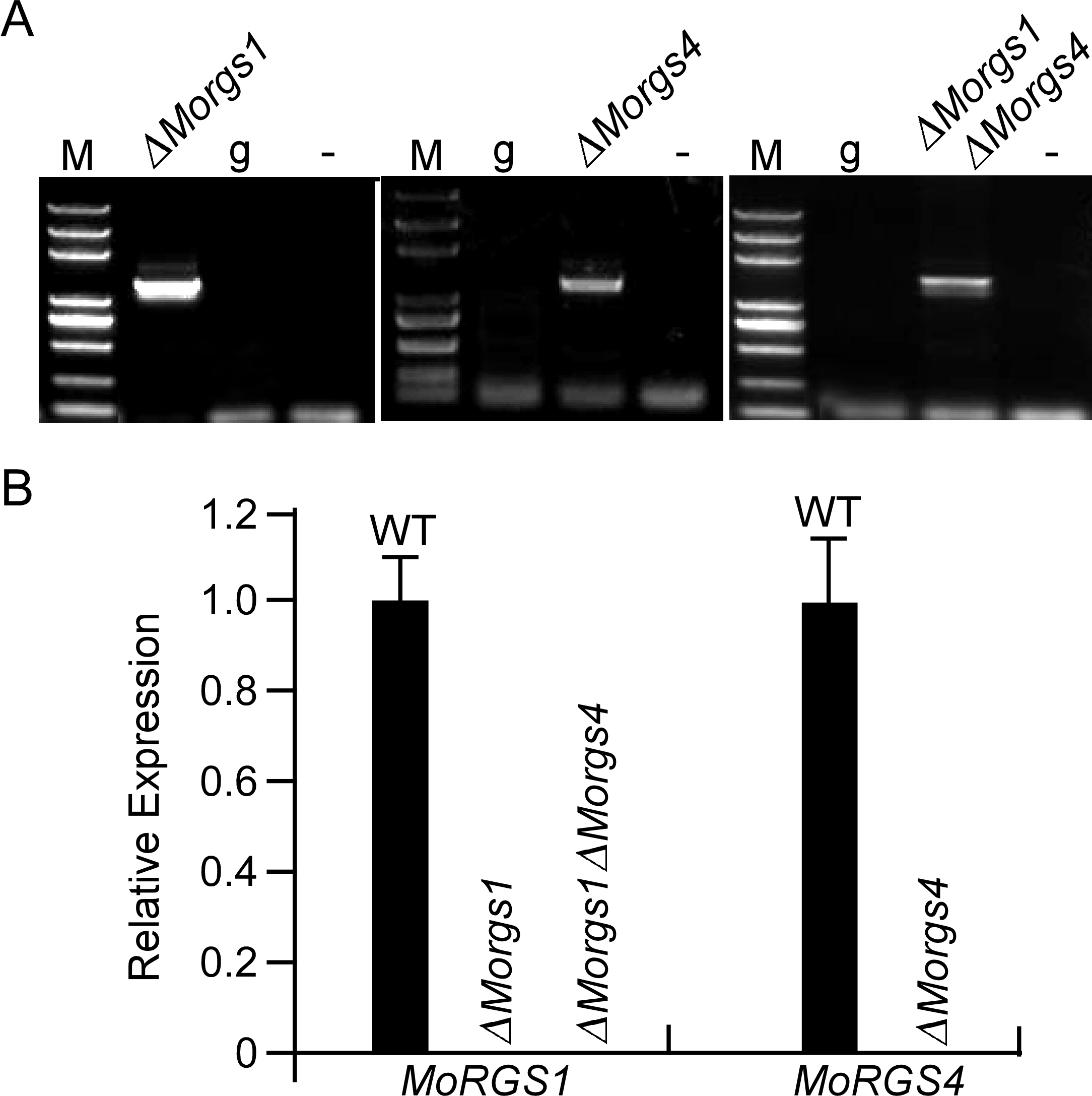

Supplement: Figure S2 — Confirmation of target gene replacement. (A) Verification mutants by PCR with one primer from resistant gene (hygromycin/ bleomycin- resistant) and one primer beyond gene flanking sequence. M, 2000 bp plus marker; g, genomic DNA; -, negative control. (B) Mutants further confirmed by qRT-PCR. ΔMorgs1ΔMorgs4 double mutant was obtained by deletion MoRGS1 in ΔMorgs4 background. (TIF) [file ppat.1002450.s002.tif]
