# Supplementary material for: Eight RGS and RGS-like Proteins Orchestrate Growth, Differentiation, and Pathogenicity of Magnaporthe oryzae
Source: PLoS Pathog. 2011 Dec 29;7(12):e1002450. doi: 10.1371/journal.ppat.1002450 (PMC3248559; doi:10.1371/journal.ppat.1002450)
Supplement: Table S1 — Primers used in this study. (DOC) [file ppat.1002450.s004.doc]

**Table S1**  Primers used in this study

| Primer | Sequence (5’-3’) | Application |
| --- | --- | --- |
| FL919 | ttgcctggttcagctagcacatgg | amplify *MoRGS1* 5’ flank sequence |
| FL1019 | tcacaaactcctcgacatccagatatcgagcgtatatttcgggattccg | amplify *MoRGS1* 5’ flank sequence |
| FL1020 | cggaatcccgaaatatacgctcgatatctggatgtcgaggagtttgtga | amplify *MoRGS1* 3’ flank sequence |
| FL920 | cctgcgatgtcttctctatgctga | amplify *MoRGS1* 3’ flank sequence |
| FL481 | agtcctggacagaattcatgccc | amplify *MoRGS2* 5’ flank sequence |
| FL482 | tgatcatggaacggacggttgcacccgggcttgaaaacacaaccttctcggg | amplify *MoRGS2* 5’ flank sequence |
| FL483 | cccgagaaggttgtgttttcaagcccgggtgcaaccgtccgttccatgatca | amplify *MoRGS2* 3’ flank sequence |
| FL484 | gagaagagaccaagatcagactc | amplify *MoRGS2* 3’ flank sequence |
| FL433 | ttcgcaatcgacaaatgggcaag | amplify *MoRGS3* 5’ flank sequence |
| FL434 | gtcgcctcgtatttctcgcgacccgggctcgatcgtgaattgcaagctat | amplify *MoRGS3* 5’ flank sequence |
| FL435 | atagcttgcaattcacgatcgagcccgggtcgcgagaaatacgaggcgac | amplify *MoRGS3* 3’ flank sequence |
| FL436 | tcacggaatcgtgcgtgtctc | amplify *MoRGS3* 3’ flank sequence |
| FL429 | aatatgatatccctgtatgccac | amplify *MoRGS4* 5’ flank sequence |
| FL430 | cgactgattacctatcatcaatcccgggggtgcagaacacaggacta | amplify *MoRGS4* 5’ flank sequence |
| FL431 | gtagtcctgtgttctgcacccccgggattgatgataggtaatcagtcg | amplify *MoRGS4* 3’ flank sequence |
| FL432 | caccagagggtcggggtc | amplify *MoRGS4* 3’ flank sequence |
| FL6835 | cttccaccatgtcgtgcggtat | amplify *MoRGS5* 5’ flank sequence |
| FL6836 | actccagacccaatgatatcggtttaaactagctctcgttgtcatgtgcg | amplify *MoRGS5* 5’ flank sequence |
| FL6837 | ccgcacatgacaacgagagctagtttaaaccgatatcattgggtctggagt | amplify *MoRGS5* 3’ flank sequence |
| FL6838 | aaggaagtcgtagatgctcgcc | amplify *MoRGS5* 3’ flank sequence |
| FL7704 | tcacgttcgtaagcctgatagg | amplify *MoRGS6* 5’ flank sequence |
| FL7705 | ttggaagacagtaatccggacgtttaaactccgtcaagcgtcgacaagatg | amplify *MoRGS6* 5’ flank sequence |
| FL7706 | catcttgtcgacgcttgacggagtttaaacgtccggattactgtcttccaa | amplify *MoRGS6* 3’ flank sequence |
| FL7707 | cgacgagcaacagccaaa | amplify *MoRGS6* 3’ flank sequence |
| FL8430 | tgagccttggcctatctttg | amplify *MoRGS7* 5’ flank sequence |
| FL8431 | tacatgccacttgcgtatccgatatcccatgtttgcttgtgtttgg | amplify *MoRGS7* 5’ flank sequence |
| FL8432 | ccaaacacaagcaaacatgggatatcggatacgcaagtggcatgta | amplify *MoRGS7* 3’ flank sequence |
| FL8433 | ctgccgcaggtttagaaaag | amplify *MoRGS7* 3’ flank sequence |
| FL8439 | attcatcaggggcttgtcag | amplify *MoRGS8* 5’ flank sequence |
| FL8440 | tgaggaaatgtaccatagcagcgtttaaaacgtgaggaaggcttgag | amplify *MoRGS8* 5’ flank sequence |
| FL8441 | ctcaagccttcctcacgttttaaacgctgctatggtacatttcctca | amplify *MoRGS8* 3’ flank sequence |
| FL8442 | gtggtcccgcgtgaagag | amplify *MoRGS8* 3’ flank sequence |
| FL1111 | ggaggtcaacacatcaatg | amplify *HPH* cassette |
| FL1112 | tcatcactctattcctttgccctcg | amplify *HPH* cassette |
| FL9949 | ggtacctgaaggagcatt | amplify bleomycin cassette |
| FL9950 | atgagctgtatctggaag | amplify bleomycin cassette |
| FL3672 | tcgagattactccgcatgacg | amplify *MoRGS1* probe sequence |
| FL3673 | acctcaatcgtatggaagtcc | amplify *MoRGS1* probe sequence |
| FL393 | atgtcatcgagctcgcggcc | amplify *MoRGS2* probe sequence |
| FL2298 | gtcggttgtgacatgacctgt | amplify *MoRGS2* probe sequence |
| FL391 | atggccgattcgatgtcaggc | amplify *MoRGS3* probe sequence |
| FL392 | ctaaagcctcttgccgggcac | amplify *MoRGS3* probe sequence |
| FL2296 | tcgaccaggaacctatgacag | amplify *MoRGS4* probe sequence |
| FL2297 | cagcaagagtagctcacgcat | amplify *MoRGS4* probe sequence |
| FL6839 | gcgagatcgacgactgagctt | amplify *MoRGS5* probe sequence |
| FL6840 | cacaacctggtagatgttggg | amplify *MoRGS5* probe sequence |
| FL6845 | ccaccaacgattactgacatgc | amplify *MoRGS6* probe sequence |
| FL6846 | acgttgagttccttctcggac | amplify *MoRGS6* probe sequence |
| FL8436 | caaataccacccatccttcg | amplify *MoRGS7* probe sequence |
| FL8437 | gtgaagaggctttcggactg | amplify *MoRGS7* probe sequence |
| FL8445 | cgcaaataccatccttcgtt | amplify *MoRGS8* probe sequence |
| FL8446 | gatttgtgcatgaccacctg | amplify *MoRGS8* probe sequence |
| FL1018 | taagaattcagtctagagtataactcattggtac | *MoRGS1* complementation |
| FL1019 | taagcggccgctcataaccgttgcgagcggctt | *MoRGS1* complementation |
| FL1067 | taatctagaatgcacgccacctcccgtctgt | *MoRGS2* complementation |
| FL1068 | taaaagctttcatgtgtgtccgatgctgaaaaa | *MoRGS2* complementation |
| FL1069 | taagaattcagatgcgcacacggatacggt | *MoRGS3* complementation |
| FL1070 | taagcggccgcctaaagcctcttgccgggcac | *MoRGS3* complementation |
| FL1051 | taaactagttgatatccctgtatgccacatcag | *MoRGS4* complementation |
| FL1052 | taatctagagtcaccaagtctggccaacaacaa | *MoRGS4* complementation |
| FL7819 | taagaattcgcatcagattacggtgaatctc | *MoRGS5* complementation |
| FL7820 | taagcggccgcctactcgccaagcatagctcc | *MoRGS5* complementation |
| FL7751 | taaatcgatcacgtggtcatgatgcagtcat | *MoRGS6* complementation |
| FL7752 | taaactagttcatccccgatgtacattctc | *MoRGS6* complementation |
| FL8651 | actcactatagggcgaattgggtactcaaattggttagacccaggattgggttcatgtc | *MoRGS7* complementation |
| FL8652 | caccaccccggtgaacagctcctcgcccttgctcacaagggcagccccgccgaac | *MoRGS7* complementation |
| FL8653 | actcactatagggcgaattgggtactcaaattggttcaaggtaggcatctagttgccgat | *MoRGS8* complementation |
| FL8654 | caccaccccggtgaacagctcctcgcccttgctcacgtacctcatgttcctcaggtacac | *MoRGS8* complementation |
| FL8641 | actcactatagggcgaattgggtactcaaattggttatgcacgccacctcccgtctgt | *MoRGS2-GFP* fusion |
| FL8642 | caccaccccggtgaacagctcctcgcccttgctcactgtgtgtccgatgctgaaaaaacg | *MoRGS2-GFP* fusion |
| FL8655 | ctatagggcgaattgggtactcaaattggttacgacctgagctgtgtgcactg | *MoMAGB-3xFLAG* fusion |
| FL8656 | ctttataatcaccgtcatggtctttgtagtcgatcagaccacagagacggaggttc | *MoMAGB-3xFLAG* fusion |
| FL4887 | gaattccgcatggacaagatcctgaac | amplify *MoRGS1* RGS domain |
| FL4888 | ctcgagttagagtagatgctcgtacctgcg | amplify *MoRGS1* RGS domain |
| FL3873 | gaattcatgtcatcgagctcgcggc | amplify *MoRGS2* cDNA sequence |
| FL3876 | atcgattcatgtgtgtccgatgctg | amplify *MoRGS2* cDNA sequence |
| FL3827 | gaattcatggccgattcgatgtcaggc | amplify *MoRGS3* cDNA sequence |
| FL3872 | ctcgagaagcctcttgccgggcaccg | amplify *MoRGS3* cDNA sequence |
| FL4885 | gaattctccctggaggaattgctcc | amplify *MoRGS4* RGS domain |
| FL4886 | ctcgagtcaaagacacttgtaaaacaagtc | amplify *MoRGS4* RGS domain |
| FL7817 | catatgatgatctaccaggttgtgctgc | amplify *MoRGS5* RGS domain |
| FL7818 | gaattcttaaagctggtgtcgaacaaag | amplify *MoRGS5* RGS domain |
| FL7753 | catatgatggccgacaccctagcacacat | amplify *MoRGS6* RGS domain |
| FL7754 | ggatccttagtaggtgccgttggacacg | amplify *MoRGS6* RGS domain |
| FL9054 | taacatatgatggcgctcgactacatgctc | amplify *MoRGS7* RGS domain |
| FL9055 | taaggatccttaagacatactataccgccc | amplify *MoRGS7* RGS domain |
| FL9056 | taagaattcatggcgctcgactacgtgct | amplify *MoRGS8* RGS domain |
| FL9057 | taagtcgacttacttgacaaactttggcc | amplify *MoRGS8* RGS domain |
| FL9963 | actcactatagggcgaattgggtactcaaattggttacgacctgagctgtgtgcactg | amplify *MoMAGB*^G183S^, *MoMAGB*^Q204L^ allele |
| FL9964 | caccaccccggtgaacagctcctcgcccttgctcacgatcagaccacagagacggaggttc | amplify *MoMAGB*^G183S^, *MoMAGB*^G183S^ allele |
| FL9965 | gctggttgtcttgacgcgagatcgaag | amplify *MoMAGB*^G183S^ allele |
| FL9966 | acgtgcttcgatctcgcgtcaagacaaccagcatcactgagacgactttcatcatt | amplify *MoMAGB*^G183S^ allele |
| FL9967 | gaggccaccaacatcgaacattcgg | amplify *MoMAGB*^Q204L^ allele |
| FL9968 | acctaccgaatgttcgatgttggtggcctccgttctgagcgaaagaagtggatt | amplify *MoMAGB*^Q204L^ allele |
| FL698 | gaattcatgggcgcttgcatgagtgcgagc | amplify *MoMAGA* cDNA sequence |
| FL699 | ctgcaggttacagaatacctgagtccttcag | amplify *MoMAGA* cDNA sequence |
| FL700 | gaattcatgggttgcggaatgagcacggag | amplify *MoMAGB* cDNA sequence |
| FL701 | ggatcccttagatcagaccacagagacggag | amplify *MoMAGB* cDNA sequence |
| FL702 | ggatccgtatgtgcttcggcagccgcaacg | amplify *MoMAGC* cDNA sequence |
| FL703 | ctgcaggttataaagttgtgcttgcggtg | amplify *MoMAGC* cDNA sequence |
| Actin_QF | ccatgtaccctggtctttcg | quantitative RT-PCR analysis |
| Actin_QR | ttcgagatccacatctgctg | quantitative RT-PCR analysis |
| RGS1_QF | gaggatgctatcaacaacctcg | quantitative RT-PCR analysis |
| RGS1_QR | cctcattgtgtaaacctgctg | quantitative RT-PCR analysis |
| RGS2_QF | atgttgcagacgagactggtc | quantitative RT-PCR analysis |
| RGS2_QR | gtgacacttgcttcatcctca | quantitative RT-PCR analysis |
| RGS3_QF | gctcaacatatgacactatgcc | quantitative RT-PCR analysis |
| RGS3_QR | tgacatctgcgcatcctgatc | quantitative RT-PCR analysis |
| RGS4_QF | gaccgcatactgcaagttgac | quantitative RT-PCR analysis |
| RGS4_QR | tgcagcttgccatcattgtac | quantitative RT-PCR analysis |
| RGS5_QF | tcgacactggcactgacatc | quantitative RT-PCR analysis |
| RGS5_QR | tcactcgcgacgtctacaag | quantitative RT-PCR analysis |
| RGS6_QF | cgagaatgccaatcgacgac | quantitative RT-PCR analysis |
| RGS6_QR | tggcgatgtagtgctggatg | quantitative RT-PCR analysis |
| RGS7_QF | gccagttcggtctgttgatt | quantitative RT-PCR analysis |
| RGS7_QR | gaacttgcctagcagcgtct | quantitative RT-PCR analysis |
| RGS8_QF | ccgagttccaggtcaacatt | quantitative RT-PCR analysis |
| RGS8_QR | ctccggtatctccccaaagt | quantitative RT-PCR analysis |
| MPG1_QF | gagaaggtcgtctcttgctg | quantitative RT-PCR analysis |
| MPG1_QR | tgtccgagcagaagttgttg | quantitative RT-PCR analysis |
| MHP1_QF | catcatcgccaccatcttc | quantitative RT-PCR analysis |
| MHP1_QR | ctggccacagtcgaggtt | quantitative RT-PCR analysis |
| MGG11608_QF | cgtccactgccacatcgc (Guo *et al*, 2010) | quantitative RT-PCR analysis |
| MGG11608_QR | agtcgtcctggtggaaagg (Guo *et al*, 2010) | quantitative RT-PCR analysis |
| MGG13464_QF | tgttccactgccacatcg (Guo *et al*, 2010) | quantitative RT-PCR analysis |
| MGG13464_QR | ctcagaccagagtcgtgctg (Guo *et al*, 2010) | quantitative RT-PCR analysis |
| MGG08200_QF | tactttgacgacgccatgac (Guo *et al*, 2010) | quantitative RT-PCR analysis |
| MGG08200_QR | cccgacggtatgtgctaagt (Guo *et al*, 2010) | quantitative RT-PCR analysis |
| MGG07790_QF | gtacgcgcagtattccaggt (Guo *et al*, 2010) | quantitative RT-PCR analysis |
| MGG07790_QR | ggtcttgttgcggaacatct(Guo *et al*, 2010) | quantitative RT-PCR analysis |
| MGG01924_QF | tccctggactgctcaagtct (Song *et al*, 2010) | quantitative RT-PCR analysis |
| MGG01924_QR | gctgtcaagaggacggtagc (Song *et al*, 2010) | quantitative RT-PCR analysis |
| MGG13291_QF | ctgggctggagcttctacac (Guo *et al*, 2010) | quantitative RT-PCR analysis |
| MGG13291_QR | gaagtcgctgtcccagtagg (Guo *et al*, 2010) | quantitative RT-PCR analysis |
| MGG11856_QF | ctcaacgacctcaacaagca (Guo *et al*, 2010) | quantitative RT-PCR analysis |
| MGG11856_QR | ggttgaactttgggttgacg (Guo *et al*, 2010) | quantitative RT-PCR analysis |
