# Supplementary material for: Eight RGS and RGS-like Proteins Orchestrate Growth, Differentiation, and Pathogenicity of Magnaporthe oryzae
Source: PLoS Pathog. 2011 Dec 29;7(12):e1002450. doi: 10.1371/journal.ppat.1002450 (PMC3248559; doi:10.1371/journal.ppat.1002450)
Supplement: Table S2 — Gene deletion mutant phenotype comparison with wild type Guy11. (DOC) [file ppat.1002450.s005.doc]

**Table S2 Gene deletion mutant phenotype comparison with wild type Guy11.**

| Strain | Protein structure | Growth | Autolysis | Surface hydrophobicity defect | Conidiation | Multi-appressorium formation on hydrophobic surface | Appressorium formation on hydrophilic surface | Penetration | Virulence | Mating | cAMP level | Laccase activity | Peroxidase activity |
| --- | --- | --- | --- | --- | --- | --- | --- | --- | --- | --- | --- | --- | --- |
|  |  |  |  |  |  |  |  |  |  |  |  |  |  |
| Guy11 |  | **+ +** | **-** | **-** | **+ + +** | **-** | **-** | **+ + +** | **+ +** | **+ +** | **+** | **+** | **+** |
| ΔMoRgs1 | 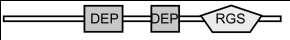 | **+** | **+** | **+** | **+ +** | **+** | **+** | **+ +** | **+** | **-** | **+ + + +** | **+** | **+** |
| ΔMoRgs2 | 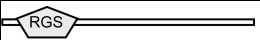 | **+ +** | **-** | **-** | **+ + + +** | **+** | **-** | **+ + +** | **+ +** | **+ +** | **+ + + + +** | **+** | **+** |
| ΔMoRgs3 | 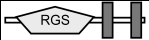 | **+ +** | **-** | **-** | **+ + + +** | **+** | **-** | **+ +** | **+** | **+ +** | **+ + +** | **+** | **+** |
| ΔMoRgs4 | 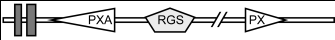 | **+** | **-** | **+** | **+** | **+** | **-** | **+ + +** | **+** | **+** | **+ + +** | **-** | **-** |
| ΔMoRgs5 | 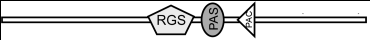 | **+ +** | **-** | **-** | **+ + +** | **-** | **-** | **+ + +** | **+ +** | **+ +** | **+ +** | **+** | **+** |
| ΔMoRgs6 | 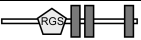 | **+ +** | **-** | **-** | **+ + +** | **+** | **-** | **+ + +** | **+ +** | **+ +** | **+ +** | **+** | **+** |
| ΔMoRgs7 | 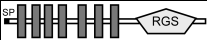 | **+ +** | **-** | **-** | **+ + +** | **+** | **-** | **+** | **+** | **+ +** | **+ + +** | **+** | **+** |
| ΔMoRgs8 | 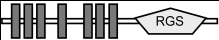 | **+ +** | **-** | **-** | **+ + +** | **-** | **-** | **+ + +** | **+ +** | **+ +** | **+ + +** | **+** | **+** |
